# Systematic review of the economic impact of novel *Mycobacterium tuberculosis* specific antigen-based skin tests for detection of TB infection compared with tuberculin skin test and interferon-gamma release assays

**DOI:** 10.1371/journal.pgph.0003655

**Published:** 2024-10-14

**Authors:** Lara Goscé, Kasim Allel, Yohhei Hamada, Elena Surkova, Irina Kontsevaya, Ting Ting Wang, Wan-Hsin Liu, Alexander Matveev, Liliya Eugenevna Ziganshina, Alexei Korobitsyn, Nazir Ismail, Saima Bashir, Claudia M. Denkinger, Ibrahim Abubakar, Peter J. White, Molebogeng X. Rangaka

**Affiliations:** 1 Institute for Global Health, University College London, London, United Kingdom; 2 Department of Infectious Disease Epidemiology, TB Modelling Group, London School of Hygiene and Tropical Medicine, London, United Kingdom; 3 Department of Disease Control, London School of Hygiene and Tropical Medicine, London, United Kingdom; 4 Royal Brompton Hospital, Part of Guy’s and St Thomas’ NHS Foundation Trust London, London, United Kingdom; 5 Division of Clinical Infectious Diseases, Research Center Borstel, Borstel, Germany; 6 German Center for Infection Research, Braunschweig, Germany; 7 Respiratory Medicine and International Health, University of Lübeck, Lübeck, Germany; 8 Faculty of Medicine, Department of Infectious Disease, Imperial College London, London, United Kingdom; 9 Department of Pediatrics, Taipei Veterans General Hospital, Taipei, Taiwan; 10 Department of Clinical Pharmacology and Therapy Named After Acad. B. Ye. Votchal, Russian Medical Academy of Continuous Professional Education, Moscow, Russian Federation; 11 Cochrane Russia, Centre for Knowledge Translation, Russian Medical Academy for Continuing Professional Education of the Ministry of Health, Moscow, Russian Federation; 12 Department of Pharmacology, Kazan Medical University, Kazan, Russian Federation; 13 Department of General and Clinical Pharmacology, RUDN University, Moscow, Russian Federation; 14 Unit for Prevention, Diagnosis, Treatment, Care and Innovation, Global Tuberculosis Programme, World Health Organization, Geneva, Switzerland; 15 Division of Infectious Diseases and Tropical Medicine at University Hospital Heidelberg Heidelberg, Baden-Württemberg, Germany; 16 Faculty of Medicine, MRC Centre for Global Infectious Disease Analysis and NIHR Health Protection Research Unit in Modelling and Health Economics, School of Public Health, Imperial College, London, United Kingdom; University of Ottawa, CANADA

## Abstract

The Purified Protein Derivative tuberculin skin tests (TST) and blood-based *Mycobacterium tuberculosis (M*.*tb)* specific interferon-gamma release assays (IGRA) are the currently used tests for identifying individuals with TB infection for preventive treatment. However, challenges around access and implementation have limited their use. Novel *M*.*tb* specific skin tests (TBST) such as Diaskintest, ESAT6-CFP10 (C-TST), C-Tb (also known as Cy-Tb), and DPPD may provide accurate and scalable options but evidence synthesis on their economic impact is lacking. We conducted two separate systematic reviews to compare the costs and cost-effectiveness of (1) the novel skin tests TBST (primary), and (2) TST and IGRA tests (secondary), to support WHO guideline development. We searched for articles presenting economic evaluations of the diagnostic tests using a health provider perspective and related to TB infection in humans. We considered papers written in English, Chinese or Russian. In the primary review, eight studies for novel TBST were found. One study in Brazil assessed cost-effectiveness of C-TST and Diaskintest and seven in Russia assessed the Diaskintest, while none evaluated C-Tb or DPPD. The review showed on average, Diaskintest kit costs (in 2021 USD) $1.60 (1.50 – 1.70), while full unit costs were estimated at $5.07. C-TST unit cost was $9.96. The second review found 32 articles on IGRA and/or the TST. These presented an average TST full unit cost of $37.88, and $87.81 for IGRA. Studies’ quality for TBST was limited while high-quality studies were found for TST and IGRA tests. In conclusion, there is limited evidence regarding the costs and cost-effectiveness of novel TBST. Conversely, there is substantial evidence for TST and IGRA tests, but most studies were performed in high-income and low-TB burden settings and their cost-effectiveness varied between and within risk groups without clear economic consensus.

## Introduction

With an estimated 1.7 billion people possibly infected with tuberculosis, progression of some infections to active TB disease poses a large public health risk [1,2]. Prominent risk factors of this transition include clinical risk factors such as human immunodeficiency virus (HIV), diabetes, undernutrition, and contextual or societal risk factors in the most vulnerable and disadvantaged populations including, TB case contacts especially children, immigrants [3]. The World Health Organization (WHO) recommends TB preventive treatment (TPT) to disrupt disease progression [4]. Testing for TB infection (TBI) is recommended to guide preventive treatment where possible. However, access and affordability of tests often present as barriers. Efficient and affordable tests for TBI are thus necessary in the effort to halt progression and the spread of TB [3].

Different strategies and tests are currently used to identify TBI. They are the tuberculin skin tests (TST) based on non-specific mycobacterial antigens, purified protein derivative (PPD), and the blood-based RD1-specific interferon-gamma release assays (IGRA). Even though both tests are useful in the control of TB, they have different implementation challenges. The TST currently requires two clinical visits within 2–3 days of testing which might be expensive and unfeasible for those individuals having limited access to healthcare, resulting in incomplete processes (failure to assess responses within allotted period invalidates results). Also, the TST can cross-react with previous Bacillus Calmette-Guérin (BCG) vaccination and non-tuberculous mycobacteria [5]. IGRA test, on the other hand, is rapidly read through the use of blood samples, requires one clinical visit for testing and recording and reporting of results can be easily integrated with laboratory information management systems. Moreover, IGRA has higher specificity in BCG vaccinated individuals as RD1 antigens are not present in BCG [6,7]. However, IGRA can be an expensive platform to set-up and maintain and assays require trained laboratory personnel to execute. Notwithstanding, neither test can accurately distinguish between TB infection and active TB disease [8], and current guidance from WHO is that either can be used in TBI testing and treatment algorithms. Moreover, due to access and implementation challenges of these tests, WHO recommends TPT without previous testing in select high-risk groups in high burden settings.

Newer *Mycobacterium tuberculosis* antigen-based skin tests (TBST) comprising specific TB antigens are now available, these include tests such as the C-Tb (now also called Cy-Tb; Serum Institute of India, India), Diaskintest (Generium, Russia), and C-TST (*nee* ESAT6-CFP10 or ECskintest; Anhui Zhifei Longcom, China). These specific TBST are suggested to be more accurate than the TST based on PPD and may offer an affordable alternative to IGRA tests. These new tests work by using a complex of recombinant proteins in a similar way to IGRA, with a recent systematic literature review and meta-analysis by Krutikov *et al* on the diagnostic performance of TBST showing that novel skin tests perform similarly to IGRA and TST in different populations and settings [9]. Clinical trials have also shown that novel skin tests have higher specificity and sensitivity for TBI, especially in resource-constrained settings and contexts where BCG vaccination is implemented routinely [9,10]. The diagnostic accuracy of the tests alone, however, is not sufficient evidence for the recommendation of their use in TBI testing guidelines. To enable policy and clinical recommendations, costs and cost-effectiveness must also be considered to inform affordability and feasibility of implementation.

The present study provides a systematic review of the literature on the costs and cost-effectiveness of the new tests for TBI (Diaskintest and C-TST). We summarise the resource considerations and costs of implementing these tests. The study also presents a systematic review of currently available tests (TST and IGRA) to allow comparison with new tests by assessing their incremental costs.

## Material and methods

We performed two systematic literature reviews to inform the development of WHO guidelines [11]. We used a combined approach to analyse the costs and cost-effectiveness of the different tests, and breakdown of the unit costs whenever possible. First, a primary review to evaluate the costs and cost-effectiveness of the novel skin tests (registered on PROSPERO: CRD42021275585). Second, since we anticipated limited data on the cost and cost-effectiveness of new skin tests, we conducted a secondary review for the costs and cost-effectiveness of TST and IGRA tests (registered on PROSPERO: CRD42021275684). This was meant to supplement the primary review to allow comparison between tests. We selected TST and IGRA tests as comparative tests given that their operational and logistic requirements are similar to those of TBST.

### Search strategy and data sources

The search for the primary and secondary review was conducted on 30 July 2021 and 20 August 2021 respectively. Following the WHO guideline development group meeting, the search was updated until 22 November 2022 for the primary review and 9 January 2023 for the second review. The databases Medline (OVID), Embase (OVID), Chinese biomedical literature, China National knowledge Infrastructure, and Russian eLibrary were used to perform the literature searches. The search strategy for the primary and secondary reviews was to split the keywords into three key concepts: (1) “Tuberculosis”, (2) “Diagnostic Test" and (3) “Cost-effectiveness”. Full search strategies used per systematic review are shown in Table A and Table B in S1 Text. We also searched articles shared by test manufacturers and those identified through a WHO’s recent public call [12]. We explored previously defined tests costs and effectiveness for TBI tests in humans, without restricting the search to any specific population group. For the primary review we searched for all articles written in Chinese, English, or Russian (Russia and China being the locations where Diaskintest and C-TST respective manufacturers are based). For the secondary review we searched for articles written in English. No time restriction was imposed for the primary review on novel skin tests, whereas we searched those articles published from July 2011 for the secondary review.

### Study selection & data extraction

Following the PRISMA guidelines [13], studies were included if they provided any economic evidence related to the tests or test implementation costs of TB infection for the following products; novel TBST (Diaskintest, C-Tb, EC skin test, DPPD), and TST or IGRA (QuantiFERON-TB Gold In-Tube /Gold Plus/ T-SPOT.TB). Only full original economic evaluations presenting costs measures, outcomes or incremental analyses using the healthcare perspective were analysed. We removed conference abstracts, reviews, letters, and opinion pieces. We screened articles titles and abstracts and full articles consecutively by involving at least two reviewers to apply our inclusion and exclusion criteria. We performed double data extraction for those articles included and they were reviewed and confirmed by native speakers. All disagreements were resolved by discussion.

We extracted studies’ data including their title, authors, interventions evaluated (test types and regime), and study’s year and population. We also extracted information on their methods’ specifications, such as the analytical model type, time horizon, discount rate, and measures of effectiveness. We recorded the results of the base-case analysis whenever the incremental cost-effectiveness ratios (ICER) were reported, alongside with the “baseline” intervention used. Studies’ sensitivity analyses estimates, and their respective variations thresholds over the average estimations, were gathered if reported. Cost components and unit test costs were obtained from the studies together with key costing input parameters. We classified studies costs into test kits/drug, staff time (nurse), consumables (syringes, gloves, etc.), equipment (fridge storage, laboratory), and overheads. All the cost estimates are inflated for the year 2021 USD (United States Dollars), and where the year of the analysis was not mentioned it was estimated from the year of publication. We assessed all included articles using Drummond’s checklist for healthcare economic evaluations to examine study quality [14]. The checklist comprises a list of ten items designed to guide critique of economic appraisals by understanding whether essential economic evaluation aspects are present or absent.

All data extraction was performed in Microsoft Excel, version 16.6, 2022.

## Results

### Identification, screening, eligibility, and inclusion

**Fig 1:**
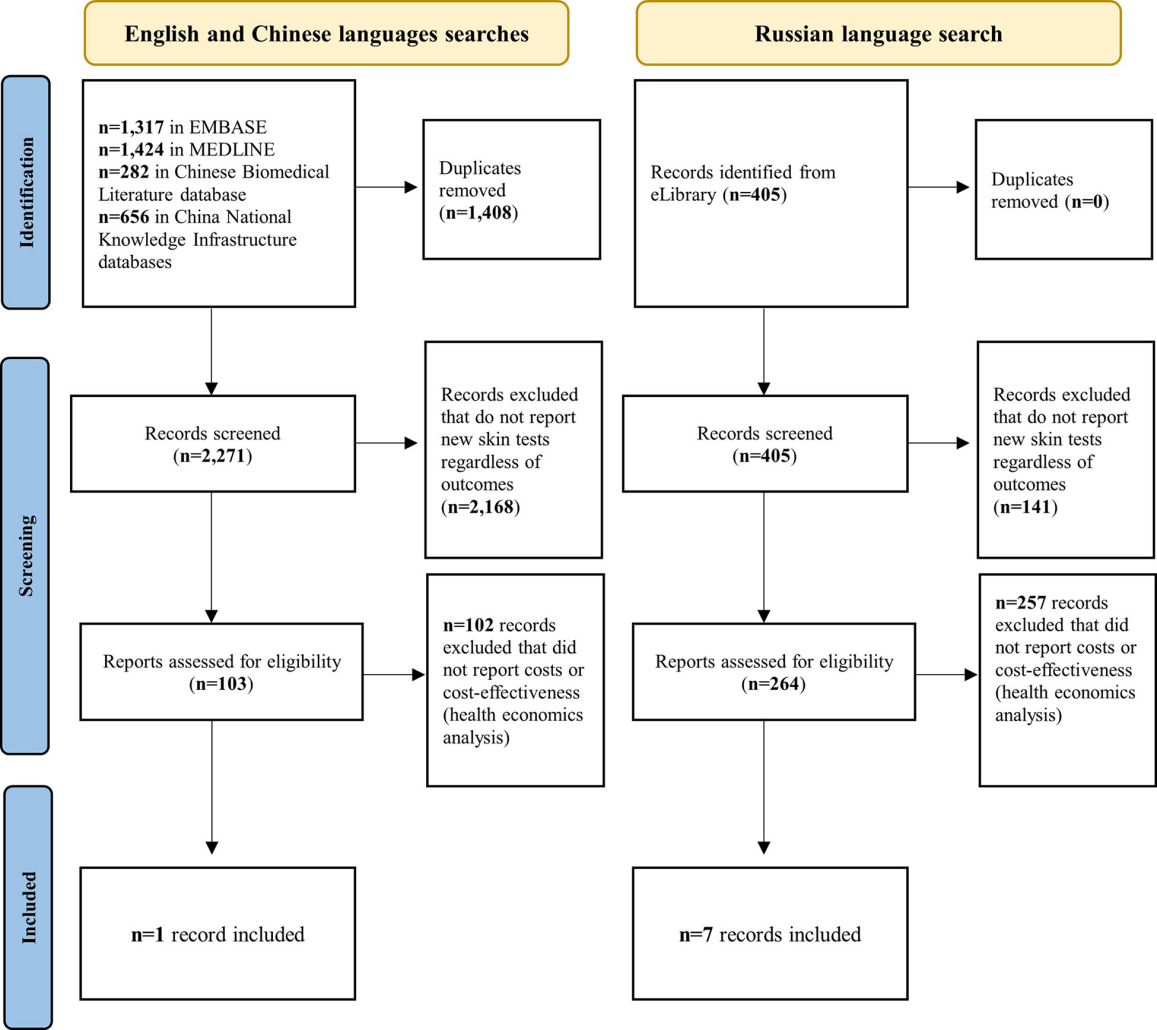
PRISMA flowchart for primary systematic review on costs and cost-effectiveness related to the novel skin test (TBST). Notes: No eligible study was identified among papers shared by test manufacturers and those identified though a WHO public call for data.

**Fig 2:**
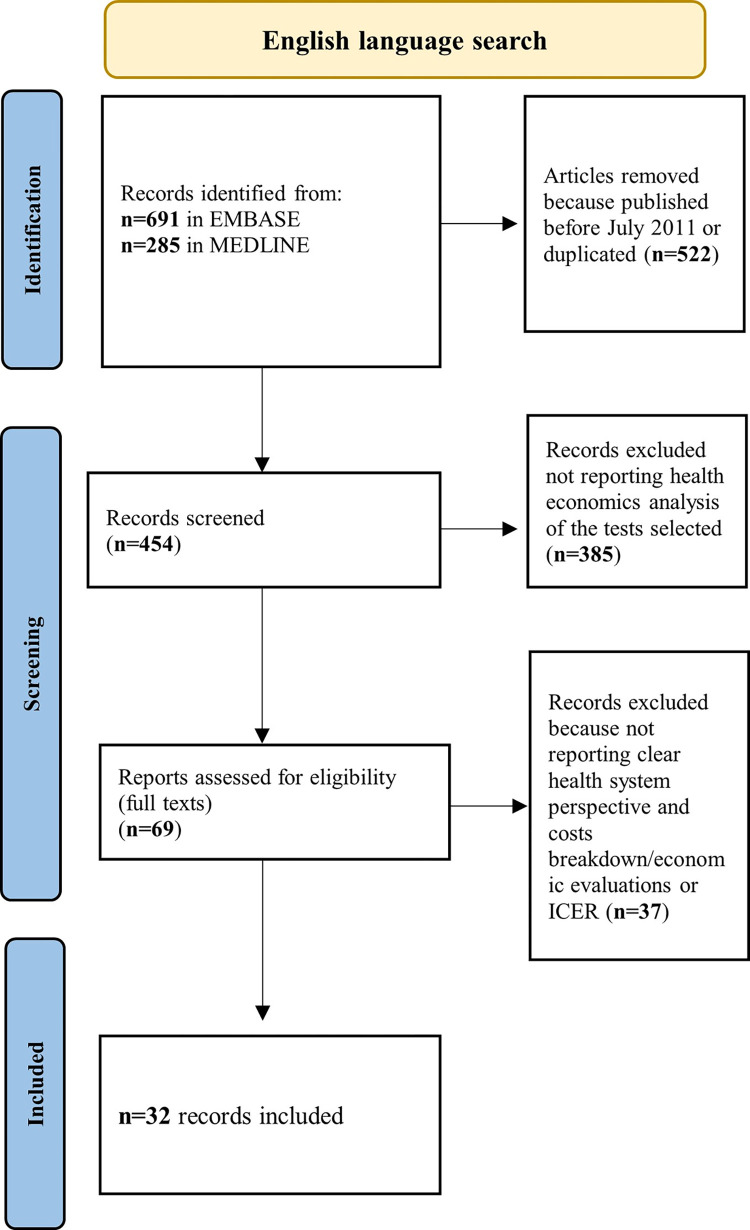
PRISMA flowchart for the secondary systematic review on costs and cost-effectiveness related to TST and IGRA. Notes: ICER: Incremental cost-effectiveness ratio.

For the primary search on cost-effectiveness of novel skin tests, 367 records were identified for full text screening (103 written in English/Chinese and 264 in Russian) of which only 8 were relevant to the research question (only one written in English and seven in Russian) (Fig 1). For the secondary search on cost-effectiveness of TST or IGRA tests, 56 out of 407 records were chosen for full-text screening and 32 papers were selected and fully included in our analyses (Fig 2). A table summarising key findings can be found in the supplementary document (Table C in S1 Text).

### Description of the articles on novel TBST

Eight economic evaluation studies were conducted for middle-income countries (Russia and Brazil), these studies mostly compared Diaskintest against TST (Table D in S1 Text). One study met most of the items on Drummond’s checklist and reported all the required information. This study, by Steffen *et al* (2020) [15] studied the cost-effectiveness of two novel TBST (Diaskintest and C-TST) for people living with HIV (PLHIV) in Brazil compared to TST and QFT-GIT. A Markov model was used to compare single screening strategies of the respective tests. The primary outcome used was incremental cost-effectiveness ratio (ICER) per incremental gain in quality-adjusted life years (QALY).

The rest of the studies were performed in Russia and were primarily focused on Diaskintest used as an alternative strategy to TST to diagnose TB infection in children. Most of these studies included cost-effectiveness analyses using the ICER (per case averted or diagnosed) as a measure of effectiveness. Studies’ quality is limited. Only 3 Russian studies specified the type of model used (decision tree) while the remaining did not. These studies did not present or provided unclear information on relevant parameters such as time horizon, type of model used, year of the evaluation, and discounting rate. Four studies did not perform any sensitivity analysis, while remaining studies only performed some form of univariate analysis (Table F in S1 Text).

### Description of the articles found evaluating the TST and IGRA tests

Of the 32 papers included in the review, nine were from the United Kingdom (UK), seven from United States of America (USA), five from Canada, two from Brazil, two from South Africa, and one each from Germany, Hong Kong, Japan, Norway, Oman, Singapore, and Spain as shown in Table E in S1 Text. The studied interventions were wide-ranging (including single and dual testing). Most studies (24) used the Quality Adjusted Life Years (QALYs) or number of cases of active TB averted as primary outcomes. Cost-utility analyses (CUA) and cost-effectiveness analyses (CEA) were often employed, and effectiveness was mostly derived by the computation of the ICERs (i.e., incremental cost per incremental QALY gained). The methods ranged from discrete event simulation, Markov models, and decision trees. Multiple articles included either one-way or two-way deterministic or probabilistic sensitivity analysis, with a reduced number providing both type of sensitivity analysis.

The included studies were of a high quality according to Drummond’s checklist (Table G and Table H in S1 Text). Most studies included and accounted for differentials in time and uncertainty, comprised more than one alternative strategy, and discussed issues of concern given the specific target groups explored.

### Summary of cost and cost-effectiveness findings

1. *Primary Review*
  One paper on the Diaskintest and C-TST [15] was found in the English and Chinese language searches, while seven papers on Diaskintest were from the Russian language searches. No papers on C-Tb test or DPPD were identified. All papers reported strategies involving Diaskintest (and one for C-TST) as cost-effective and/or cost-saving. For unit costs, these mostly comprised of test kits/drugs, staff time, consumables, equipment used and, less commonly, overheads. However, unit costs vary by economic evaluation and some of them provide no information on the composition. The unit cost of Diaskintest was estimated as $5.07, whereas C-TST was $9.96 as per calculated by Steffen *et al*. 2020 (Table 1). Diaskintest was preferred to QFT-GIT and TST (cost saving estimate per QALY was US $1,375). In probabilistic sensitivity analysis (PSA), Steffen compared strategies to Diaskintest only. The dominance of Diaskintest was very sensitive to the cost of Diaskintest which is highly uncertain due to using market value and hence varies widely by health system and country. Two papers reported no or unclear unit costs. The rest of the articles found that the cost of the Diaskintest kit ranged from $1.29 to $3.49 and that it was not very sensitive to variation in unit costs after employing univariate sensitivity analyses (if measured). All these studies, apart from Steffen *et al*., found that Diaskintest was cost-effective using a wide-range of methods, ranging from a cost-effectiveness ratio of 2.28 times the local currency compared to 3.42 for TST to total costs saving of $757.7 for Diaskintest, compared to TST in children populations between 2009 and 2020 (Table D in S1 Text). For instance, Yagudina *et al*. 2013 [16] found the estimate of ICER was $1,666, whereas Solodun *et al*. 2017 [17] found that it was $10,586.6 for Diaskintest, being highly cost-effective, compared to TST (ICER:$49,523.9 and ICER:$40,641, respectively) (Table D in S1 Text for studies details). The difference in ICERs between these two Diaskintest studies is that Solodun *et al*. 2017 [17] included costs for chest radiography and additional tests in the costing scheme.
2. *Secondary review*

**Table 1:** Unit costs of novel TBST test, Diaskintest, resulted from the primary review.

Study ID	Country	Test	Test Kit	Staff Time	Consumable	Overheads	Equipment	Unit Costs ($, 2021)
Aksenova (2021) [18]	Russia	Diaskintest	1.70					1.70
Kulikov (2009) [19]	Russia	Diaskintest	1.50					1.50
Moiseeva (2014) [20]	Russia	Diaskintest	1.61					1.61
Solodun (2017) [17]	Russia	Diaskintest	1.60					1.60
Steffen (2020) [15]	Brazil	Diaskintest: C-TST:	1.50 6.30	2.24	1.38		0.04	5.07 9.96
Yagudina (2013) [16]	Russia	Diaskintest:						3.50

*Notes*: The costs for all screening strategies include the costs of the tests (disposables, administration, reading, laboratory technicians), two clinic visits and one chest radiograph. Costs are presented in 2021 USD. We have presented only total unit costs for those articles without information on costs components due to lack of evidence provided.

Most studies evaluated the costs and cost-effectiveness of TST and IGRA with wide-ranging study populations including PLHIV, immunocompromised people other than PLHIV, immigrants/migrants, and healthcare workers. Twenty-five studies were set in low TB burden countries (UK, USA, Canada, Norway, Oman, Spain, Germany), four in lower moderate (Brazil, Japan, Singapore), one in upper-moderate (Hong Kong) and two in a high burden country (South Africa). Four studies were based in low-and-middle income countries, whereas twenty-five in high-income countries. Most studies used decision analytic models (Markov-chain techniques). Results from the articles suggested that testing of any form (TST or IGRA) was more likely to be cost-effective, compared with no test strategy, when done for high-risk populations or higher burden contexts. However, no consensus exists about whether to utilise TST or IGRA. Of the 8 studies analysing the cost-effectiveness of TB infection screening in PLHIV, all found IGRA to be more cost-effective including one TST [21], three a combined sequential strategy of QFT+ TST [22–24], and one found IGRA to be cost-saving over TST but Diaskintest to be the most cost-effective overall [15]. For the remaining three, Jo *et al*. 2020 [25] found that an ICER of $11,000/QALY gained (New York) and as low as only $5,000/QALY (Texas) for IGRA compared to TST, whereas Capocci *et al*. 2015 [26] found that QFT was the most cost-effective strategy with an ICER of £9,332/QALY gained compared to no testing. Finally, Linas *et al*. 2011 [27] found similar results for IGRA with an ICER of $23800/QALY compared to TST.

Three studies focused on groups of immunocompromised individuals other than PLHIV, these found no testing to be the most cost-effective strategy. However, all three studies are based in low-burden countries [21,27,28]. Among healthcare workers, the five studies reported either IGRA or TST to be the most cost-effective strategy. Similarly, the five studies focusing on the screening of contacts of active TB cases, showed no consensus about whether to utilise TST or IGRA, with similar numbers being marginally more cost-effective for either one or the other alternative, or a strategy combining the two tests. All 12 studies analysing cost-effectiveness of TB infection screening in migrants in high-income countries, showed that screening with either IGRA or TST is preferred to no screening strategies. 80% of the studies comparing the two tests reported IGRA more likely to be cost-effective, one study [29] found the combined sequential TST+QFT strategy to be the most cost-effective, and one [22] found TST (>5mm cut off) to be the most cost-effective strategy compared to QFT-GIT. Tables 2 and 3 present the unit costs extracted from the secondary review by type of test. Mean unit cost of TST and IGRA were $37.88 and $87.81 respectively. High variability of staff costs, especially among high-income countries, represents the major driver of heterogeneity among unit costs from different sources; some studies included more consultations/visits (provided by medical staff rather than nurses), resulting in higher unit costs. Finally, we found greater costs for the IGRA test in South Africa due to the inclusion of chest radiography within the costing scheme (Table 3).

**Table 2:** Unit costs of TST, resulted from the secondary review.

Study ID	Country	Test Kit	Staff Time	Consumable	Overheads	Equipment	Unit Costs ($, 2021)
Loureiro (2019) [30]	Brazil	4.71	2.41	1.49		0.06	8.67
Steffen (2020) [15]	Brazil	3.99	2.24	1.38		0.04	7.66
Campbell, (2017) [43]	Canada	9.44	17.16				26.61
Campbell (2019a) [31]	Canada	9.44	17.16				26.61
Campbell, (2019b) [28]	Canada	9.44	17.16				26.61
Mullie (2017) [32]	Canada						13.51
Verma (2013) [33]	Canada	16.81	26.77				43.58
Sohn (2018) [34]	Japan	✓	✓	✓	✓		32.61
Haukaas (2017) [35]	Norway						34.24
Del Campo, (2012) [36]	Spain	✓	✓	✓			60.35
Kim (2018) [37]	South Africa	4.4	1.75	1.70	0.08		7.93
Mandalakas, (2013) ^b^ [38]	South Africa	✓	✓	✓			21.92‒99.13
Abubakar (2018) ^a^ [29]	UK	1.77	179.87 (2 clinic visits)				181.63
Auguste (2016) ^b^ [22]	UK	✓	✓	✓			27.61
Auguste (2022) ^b^ [24]	UK	✓	✓	✓			27.43
Capocci, (2015) ^c^ [26]	UK						29.60
Eralp, (2012) ^d^ [39]	UK						31.12
Pareek (2013) ^e^ [40]	UK						68.34
Fekadu (2022) [23]	USA	✓					32.45
Linas (2011) [27]	USA	2.88	23.92				26.81
Nijhawan, (2016) [41]	USA	9.38	12.73				22.10
Steffen (2013) [8]	USA	5.83	3.79	2.84		0.10	12.56
Swaminath, (2013) [49]	USA						48.99
Tasillo (2017) [21]	USA						9.06
Wingate (2015) [42]	USA						USA: 28.54 Kenya (Pre- arrival): 5.35

*Notes*: A tick mark *(✓) s*tands for those articles that mention they included certain cost components but did not explicitly state the figures and only included the total costs per test diagnostic. There were no further details provided but only the overall costs of the test If no tick (*✓)* is observed.

^a^ Staff time money values provided by the Department of Health and Social Care (DHSC). NHS Tariffs Reference Costs. London: DHSC; 2014. URL: www.gov.uk/government/collections/nhs-reference-costs.

^b^ Consumable costs referred as disposable.

^c^ Calculated from NICE. Tuberculosis—clinical diagnosis and management of tuberculosis, and measures for its prevention and control. NICE Clinical Guideline 117 2011.

^d^ Calculated from the National Institute for Health and Clinical Excellence. Clinical diagnosis and management of Tuberculosis, and measures for its prevention and control. NICE Clinical Guidelines 2011.

^e^ Calculated from the National Collaborating Centre for Chronic Conditions. Tuberculosis: A clinical diagnosis and management of tuberculosis, and measures for its prevention and control. London: Royal College of Physicians, 2011.

The costs for all screening strategies include the costs of the tests (disposables, administration, reading, laboratory technicians), two clinic visits and one chest radiograph.

Costs estimates are inflated for the year 2021 and are presented in USD.

**Table 3:** Unit costs of IGRA, resulted from the secondary review.

Study ID	Country	Test Kit	Staff Time	Consumable	Overheads	Equipment	Unit Costs ($, 2021)
Loureiro (2019) [30]	Brazil	38.54	2.55	2.06		1.22	44.36
Steffen (2020) [15]	Brazil	16.77	2.36	1.91		1.13	22.17
Campbell, (2017) [43]	Canada	40.34	6.01				46.34
Campbell (2019a) [31]	Canada	40.34	6.01				46.34
Mullie (2017) [32]	Canada						45.04
Verma (2013) [33]	Canada	33.63	26.77				60.39
Marx (2021) [44]	Germany	✓				✓	56.23
Li (2018) [45]	Hong Kong						76.10
Sohn (2018) [34]	Japan	76.25	✓	✓	✓		97.44
Haukaas (2017) [35]	Norway						76.27
Png (2019) [46]	Singapore						81.90
Kim (2018) [37]	South Africa	65.35	1.63	13.7	0.05	44	QFT: 124.73
Mandalakas, (2013)^a^ [38]	South Africa	✓	✓	✓		✓	T-SPOT: 247.12 QFT: 220.02
Del Campo (2012) [36]	Spain	✓	✓	✓		✓	65.27
Abubakar (2018)^b^ [29]	UK	T-SPOT: 102.99 QFT-GIT: 59.47	89.93 (1 clinic visit)				T-SPOT: 192.91 QFT-GIT: 149.40
Auguste (2016) [22]	UK	✓	✓	✓		✓	QFT-GIT: 76.98 T-SPOT: 55.29
Auguste (2022) [24]	UK	✓		✓			QFT-GIT: 76.48 T-SPOT: 93.53
Capocci, (2015) ^c^ [26]	UK						109.99
Eralp, (2012) ^d^ [39]	UK						87.08
Pareek, (2011) [47]	UK	✓		✓			87.05
Pareek (2013) ^e^ [40]	UK	✓		✓			QFT: 103.84 T-SP0T: 163.76
Fekadu (2022) [23]	USA	✓					QFT-GIT: 57.48 T-SPOT: 92.71
Jo (2020) [25]	USA						81.54–92.41
Linas (2011) [27]	USA						62.83
Nijhawan, (2016) [41]	USA	43.36	3.20				46.56
Shah (2012) [48]	USA	28.44	4.61	15.19	0.63	1.36	50.24
Steffen (2013) [8]	USA	51.07	1.90	2.78		1.63	57.38
Swaminath, (2013) [49]	USA						60.67
Tasillo (2017) [21]	USA						97.16

*Notes* A tick mark (✓) stands for those articles that mention they included certain cost components but did not explicitly state the figures and only included the total costs per diagnostic test. There were no further details provided but only the overall costs of the test if no tick (*✓) is observed*.

^a^ ats here referred as disposable.

^b^ Staff time money values provided by the Department of Health and Social Care (DHSC). NHS Tariffs Reference Costs. London: DHSC; 2014. URL: www.gov.uk/government/collections/nhs-reference-costs.

^c^ Calculated from NICE. Tuberculosis—clinical diagnosis and management of tuberculosis, and measures for its prevention and control. NICE Clinical Guideline 117 2011.

^d^ Calculated from the National Institute for Health and Clinical Excellence. Clinical diagnosis and management of Tuberculosis, and measures for its prevention and control. NICE Clinical Guidelines 2011

^e^ Calculated from the National Collaborating Centre for Chronic Conditions. Tuberculosis: Clinical diagnosis and management of tuberculosis, and measures for its prevention and control. London: Royal College of Physicians, 2011.

The costs for all screening strategies include the costs of the tests (disposables, administration, reading, laboratory technicians), two clinic visits and one chest radiograph. Screening strategies that include an IGRA also include the cost of one outpatient laboratory visit.

QFT: QuantiFERON-TB Gold.

Cost estimates are inflated for the year 2021 and presented in USD.

## Discussion

Our review identified studies reporting economic and cost-effectiveness analyses regarding the novel TBST, compared with TST and IGRA tests. We found 8 and 32 articles for TBST and TST or IGRA tests, respectively. Diaskintest was a frequently studied TBST followed by C-TST. Our analyses indicate that the average economic costs of TBST (Diaskintest($5.07) and C-TST($9.99)) were substantially lower than those of IGRA ($87.81, ($22.17 - $247.12)) and TST ($37.88, ($7.66 - $181.63)), after considering equal costing ingredients. We have presented the first comprehensive systematic review of currently available evidence of costs and cost effectiveness of novel TBST for tuberculosis infection in comparison with widely used TBI tests. There was, however, limited high quality studies and evidence for high-risk groups. Therefore, our results should be interpreted carefully.

A previous review on the diagnostic accuracy of TBST suggested comparability with IGRA but with the usability of the TST [50]. Estimated ICERs report that screening strategies are cost-effective among the general population using author determined willingness-to-pay (WTP) thresholds. Main drivers of economic costs were the test kit costs; Diaskintest had the lowest costs by a large margin and exceeded that of the TST and IGRA tests. Whereas main factors affecting cost-effectiveness were test’s costs, sensitivity, and specificity, in line with current literature [51]. Studies comparability was hampered by the diverging assumption on tests parameters and costs but also in modelling approaches and outcomes used. These studies also differed in the use of outcomes including net benefits and ICER per QALYs gained, DALYs or active TB case averted, or life year gained; therefore, making it difficult to contrast them.

The majority of included articles examined TST and IGRA tests’ costs and cost-effectiveness, whereas this evidence is very limited for TBST. Available TBST studies were based on two country settings (Brazil or Russia), and most focused only on Diaskintest and reported its cost-effectiveness compared to TST. This limits the interpretation and applicability of the findings, but it orientates future initiatives.

Among TST and IGRA studies, they relied primarily on high-income countries (HICs) having low- or lower-moderate TB burden. Testing strategies, compared to no testing, were always preferred among healthcare workers, immigrants, immunocompromised, and PLHIV. Yet, comparison of costs and cost-effectiveness between IGRA and TST was not always decisive.

Cost-effective strategies ranged between ICER = $50 to over $50,000 for testing healthcare workers, due to the high variability of labour costs and disease burden in different countries. Specifically, contacts of active TB cases had the largest range of TB incidence levels in our review. We found cost data from low [27] to severely endemic [38] TB, which posits further complexity when comparing cost-effectiveness among this group. Also, we encountered different conclusions/interpretations on preferred strategies when age groups were contrasted.

Younger children have a higher reactivation rate of TBI than older children [52] and hence comparison between child contacts is complicated. IGRA have not been used extensively to test for TBI in children due to limited data until recently about its performance in this population and the need for phlebotomy [53]; hence the lack of economic evidence.

Among studies in migrants or immigrants, IGRA were consistently more cost-effective than the use of TST and no screening strategies. This could be attributed to the study settings (mostly were HICs with low TB burden) which resulted in increased healthcare costs incurred by TST and greater willingness-to-pay thresholds. Evidence among immunocompromised individuals without HIV or other medical conditions found no TBI screening to be the preferred strategy due to the competing risks of death which limit life expectancy and lessen the remaining years at risk for developing TB while reducing the benefit of preventing TB [21,27].

We found studies to be wide-ranging with respect to models, testing strategies, settings, and TB burden levels, therefore reducing the generalisability of results. Nevertheless, we can make several conclusions based on the findings of this review. Firstly, we evidenced a lack of articles from LMICs, showing paucity economic evidence for where the TB burden is largest [1]. Secondly, despite no universal consensus is shown among the usage of TST or IGRA tests, results of the exhibited studies can give us important indications on the possible cost-effectiveness of TBST. In those settings where IGRA is preferred due to human resource costs (i.e. costly second visits for TST) limited benefit is likely to be applicable to TBST. Whereas in those settings where TST is less expensive, TBST can possibly bring value because of the similar accuracy to IGRA [4,11].

This study has limitations. First, there is a limited number of studies assessing TBST costs compared to TST and IGRA, which indicates inconclusive evidence for likely economic impact. Nonetheless, we provide the most updated overview of the literature and state the basis for further research. Second, the quality of existing studies for TBST is poor. For instance, some studies might bias the results of our reported costs [16–19,54] because they faced greater uncertainty according to our risk of bias assessment, none were randomized controlled trials and there was high variation over changes in parameter values. Third, we only found studies reporting either the Diaskintest or C-TST costs, and no data for C-Tb and DPPD tests were encountered. The economic performance of these tests is needed to consequently evaluate cost-effectiveness across the TBST and other test types. Nonetheless, previous estimates have observed similar performance trends between C-TST (vs DPPD) and IGRA (vs TST) tests [9]. Also, indirect comparative studies have indicated similar outcomes between all TBST tests [9].

The present systematic review has several strengths. First, we have employed two different screenings over the costs and costs-effectiveness of novel tests and most widely accepted tests for TBI using a comparative approach. Second, we searched the literature written in English, Chinese and Russian using representative datasets including national and international medical repositories. Third, our study is in line with WHO guidelines for evaluation of TBI screening tests [55]. Fourth, we compiled the information on economic techniques and methods used to evaluate different cost-effectiveness schemes. Fifth, we clearly identified where gaps in the literature exist in order to inform future investigations.

Future investigations may consider further under-represented subpopulation analyses within different healthcare settings and at-risk populations to estimate cost-effectiveness and their likely impact. Our study is relevant as it comprises important information for decision-makers, including clinicians, researchers, policy makers, and stakeholders among high and low TB burden countries. This review provides a basis for future cost-effectiveness analyses of novel tests by providing most up-to-date cost and cost-effectiveness data for TBST and current testing strategies; the TST and IGRA. Using TBST as alternative, however, must be evaluated through post-licensure studies assessing screening algorithms and their resultant outcomes on the cascade of care to help find new ways to tackle TB among different populations.

## Supporting information

S1 Checklist (DOCX)

S1 Text **Table A.** Search strategy for the primary systematic literature review. **Table B**. Search strategy for the secondary systematic literature review (TST and IGRA). **Table C**. Summary of our results/findings of both reviews. **Table D**. Data extraction results for all the articles found in the primary systematic review. **Table E**. Data extraction results for all the entries from the secondary systematic review. **Table F**. Drummond checklist for studies quality: Cost/Cost-effectiveness analyses for Novel skin tests for diagnosing TBI. **Table G**. Drummond Checklist for studies quality: Cost/Cost-effectiveness analyses for TST or IGRA for diagnosing TBI. **Table H**. Summary of the proportion of articles accomplishing each of the Drummond’s criteria.
(DOCX)

S1 Table **1**. Primary review English language. 2. Primary review Chinese language. 3. Primary review Russian language. 4. Secondary review.
(XLSX)
