# Supplementary material for: Systematic review of the economic impact of novel Mycobacterium tuberculosis specific antigen-based skin tests for detection of TB infection compared with tuberculin skin test and interferon-gamma release assays
Source: PLOS Glob Public Health. 2024 Oct 14;4(10):e0003655. doi: 10.1371/journal.pgph.0003655 (PMC11472927; doi:10.1371/journal.pgph.0003655)
Supplement: S1 Text — Table A. Search strategy for the primary systematic literature review. Table B. Search strategy for the secondary systematic literature review (TST and IGRA). Table C. Summary of our results/findings of both reviews. Table D. Data extraction results for all the articles found in the primary systematic review. Table E. Data extraction results for all the entries from the secondary systematic review. Table F. Drummond checklist for studies quality: Cost/Cost-effectiveness analyses for Novel skin tests for diagnosing TBI. Table G. Drummond Checklist for studies quality: Cost/Cost-effectiveness analyses for TST or IGRA for diagnosing TBI. Table H. Summary of the proportion of articles accomplishing each of the Drummond’s criteria. (DOCX) [file pgph.0003655.s002.docx]

**Supplementary appendix**

Systematic review of the economic impact of novel Mycobacterium tuberculosis specific antigen-based skin tests for detection of TB infection compared to tuberculin skin test and interferon-gamma release assays

Table of contents

[I. Full search strategy and main results 2](#_Toc161008502)

[II. Full data extraction of the results for all articles included in the primary review 5](#_Toc161008503)

[III. Full data extraction of the results for all articles included in the secondary review 8](#_Toc161008504)

[IV. Assessing studies quality using Drummond’s checklist for novel skin test 20](#_Toc161008505)

[V. Assessing studies quality using Drummond’s checklist for TST or IGRA tests 31](#_Toc161008506)

[VI. Summary of Drummond’s checklist 63](#_Toc161008507)

[VII. References 65](#_Toc161008508)

# **Full search strategy and main results**

For the primary systematic literature review (new skin tests), the search included papers from inception until 30 July 2021. Since the previous review included papers published until 20 October 2020, our updated search excluded studies published before October 2020.

**Table A.** Search strategy for the primary systematic literature review

|  | **Search Term** |
| --- | --- |
| 1 | exp TUBERCULOSIS/ or tuberculosis.mp. or exp MYCOBACTERIUM TUBERCULOSIS/ or tb.mp. |
| 2 | exp Recombinant Proteins/ or (recombinant or novel or dppd or esat 6 or esat6 or cfp 10 or cfp10 or early secretory antigenic target* or culture filtrate protein* or rd* or region of difference or Rv0061 or recombinant tuberculosis allergen).mp. |
| 3 | skin test*.mp. or Skin Tests/ |
| 4 | (c tb or diaskintest or c-tst or dppd).mp. |
| 5 | 1 and 2 and 3 |
| 6 | (recombinant and allergen).mp. |
| 7 | 6 and 1 |
| 8 | 4 or 5 or 7 |

**Other databases:**

e-library ([www.e-library.ru](http://www.e-library.ru)) for Russian literature

Search Terms: Диаскинтест* или «Аллерген* туберкулезн* рекомбинантн*»

The Chinese Biomedical Literature Database and the China National Knowledge Infrastructure databases

Search terms: “ESAT-6” and “CFP-10” or “ESAT6” and “CFP10”

For the secondary systematic review, we included all the articles looking at TST and IGRA cost-effectiveness since 2011.

**Table B** Search strategy for the secondary systematic literature review (TST and IGRA)

|  | **Search Term** |
| --- | --- |
| 1 | exp TUBERCULOSIS/ or tuberculosis.mp. or exp MYCOBACTERIUM  TUBERCULOSIS/ or tb.mp. |
| 2 | (TST or Tuberculin Skin Test or Tuberculin Test* or IGRA* or Interferon gamma release assay* or Interferon gamma release test*  or QFT or QFT-GIT or QuantiFERON-TB Gold In-tube or T-SPOT).mp. |
| 3 | (health care cost* or cost analysis or cost-effectiveness analysis or  cost-benefit analysis or economic evaluation or cost-utility analysis or  economic evaluation or markov model or markov chain or decision  analytic* tree*).mp. or exp COST/ or exp Cost analysis/ |
| 4 | 1 and 2 and 3 |
| 5 | limit 11 to yr="2011 -Current" |

**Table C**. Summary of our results/findings of both reviews.

| **Study, year** | **Country** | **Test** | **Study design** | **Comparator** | **Unit cost** | **Quality score (Drummond)** |
| --- | --- | --- | --- | --- | --- | --- |
| Kulikov 2009 | Russia | Diaskintest | CEA | TST | $1.50 | 5.7 |
| Aksenova 2011 | Russia | Diaskintest | Cost | TST | $1.70 | 3.2 |
| Yagudina 2013 | Russia | Diaskintest | CEA | TST | $3.50 | 5.2 |
| Moiseeva 2014 | Russia | Diaskintest | CEA | TST | $1.61 | 3.5 |
| Solodun 2017 | Russia | Diaskintest | CEA | TST | $1.60 | 4.8 |
| Sinitsyn 2018 | Russia | Diaskintest | CBA | TST |  | 4.1 |
| Chugaev 2020 | Russia | Diaskintest | Cost | TST |  | 4.4 |
| Steffen 2020 | Brazil | Diaskintest or  C-TST | CUA | TST | $5.07 $9.96 | 7.4 |
| Linas 2011 | USA | QFT | CUA | No screening | $26.81 | 6.8 |
| Pareek 2011 | UK | IGRA | CEA | No screening | $87.05 | 7.8 |
| Del Campo 2012 | Spain | TST | CEA | TST | $60.35 | 5.2 |
| Eralp. 2012 | UK | QFT or TST | CEA | TST+QFT | $87.08 (QFT) $31.12 (TST) | 6.7 |
| Shah 2012 | USA | TST + QFT-GIT | CEA | TST | $50.24 (QFT) | 6.2 |
| Mandalakas 2013 | South Africa | TST + QFT or T-SPOT, or QFT and T-spot alone | CEA | No screening | $21.92‒99.13 (TST) $220.02 (QFT) $247.12 (T-SPOT) | 7.4 |
| Pareek 2013 | UK | T-SPOT, QFT or TST+QFT or T-SPOT | CEA | TST | $68.34 (TST)  $103.84 (QFT)  $163.76 (T-SP0T) | 7.1 |
| Steffen 2013 | USA | QFT or QFT+TST | CEA | TST | $12.56 (TST) $57.38 (QFT) | 6.1 |
| Swaminath 2013 | USA | QFT | CEA | TST | $48.99 | 5.2 |
| Verma 2013 | Canada | TST | CEA | No screening | $43.58 | 6.7 |
| Capocci, 2015 | UK | TST+QFT, or QFT | CEA | No screening | $29.60 (TST) $109.99 (QFT) | 6.9 |
| Wingate 2015 | USA | TST | CBA | No screening | $28.54 | 6.1 |
| Auguste 2016 | UK | IGRA or IGRA+TST | CUA | TST | $27.61 (TST) $76.98 (QFT)  $55.29 (T-SPOT) | 6.4 |
| Nijhawan 2016 | USA | QFT | Cost | TST | $46.56 | 5.3 |
| Campbell 2017 | Canada | TST or IGRA | CUA | No screening | $26.61 (TST) $46.34 (IGRA) | 6.9 |
| Haukaas 2017 | Norway | QFT + TST or alone | CEA | No screening | $34.24 (TST) $76.27 (QFT) | 7.2 |
| Mullie 2017 | Canada | QFT-GIT | CUA | TST | $13.51 (TST)  $45.04 (QFT) | 6.6 |
| Tasillo 2017 | USA | TST, IGRA, and TST+IGRA | CUA | No screening | $9.06 (TST)  $97.16 (IGRA) | 6.1 |
| Abubakar 2018 | UK | T-SPOT, QFT, TST | CUA | No screening | $181.63 (TST) $149.40 (QFT) $192.91 (T-SPOT) | 6.7 |
| Li 2018 | Hong Kong | IGRA | CUA | No screening | $76.10 | 7.8 |
| Sohn 2018 | Japan | TST+QFT or QFT | CEA | TST | $32.61 (TST)  $97.44 (QFT) | 7.4 |
| Campbell 2019a,b | Canada | QFT or TST+QFT or TST | CUA | No screening | $26.61 (TST)  $46.34 (QFT) | 7.3 |
| Loureiro 2019 | Brazil | TST>10mm or TST+QFT | CEA | TST >5mm | $8.67 (TST)  $44.36 (QFT) | 6.4 |
| Png 2019 | Singapore | QFT | CUA | No screening | $81.90 | 7.4 |
| Al Abri 2020 | Oman | QFT or TST or CXR | CUA | QFT (4month RIF) |  | 4.4 |
| Jo 2020 | USA | IGRA | CUA | Not stated | $81.54-92.41 | 6.1 |
| Steffen 2020 | Brazil | TST or QFT-GIT or EC test | CUA | Diaskintest | $7.66 (TST)  $22.17 (QFT) | 7.4 |
| Kim 2018 | South Africa | TST | CEA | QFT-GIT | $7.93 | 7.2 |
| Auguste 2022 | UK | TST or QFT or T-SPOT or QFT-GIT 5mm | CEA | No screening | $27.43 (TST)  $76.48 (QFT)  $93.53 (T-SPOT) | 7.2 |
| Fekadu 2022 | USA | TST or T-SPOT or QFT plus | CEA | Less costly option | $32.45 (TST)  $57.48 (QFT)  $92.71 (T-SPOT) | 7.0 |
| Marx 2021 | Germany | QFT-GIT | CEA | No screening | $56.23 | 6.5 |

Notes: TST= Tuberculin skin test. CEA= Cost-effectiveness analysis. CUA= Cost-utility analysis. CBA= Cost-benefit analysis. UK= United Kingdom. USA= United States of America.

# II. Full data extraction of the results for all articles included in the primary review

**Table D:** Data extraction results for all the articles found in the primary systematic review (N=8)

| **Study Characteristics** | **Kulikov 2009 (1)** | **Aksenova 2011 (2)** | **Yagudina 2013 (3)** |
| --- | --- | --- | --- |
| **Country Setting** | Russia | Russia | Russia |
| **Year of Cost Valuation** | Not Stated | 2010 (Assumption) | Not stated. |
| **Currency** | Rubles | Rubles | Rubles |
| **Study Population** | Children and Adolescents | Children | Children and Adolescents |
| **Index Diagnostic Test Strategies** | DiaskinTest | DiaskinTest | DiaskinTest  TST + DiaskinTest |
| **Alternative Provided** | TST | TST | TST |
| **Type of economic evaluation** | CEA | Cost Analysis | CEA |
| **Source of costing** | Studies varying DiaskinTest costs in relation to ICER of Mantoux. | Empirical Data Collection | Published Literature |
| **Primary outcome** | ICER: RUB/additional TB case diagnosed | Retrospective costing. | ICER: RUB/active TB case averted. |
| **Type of model** | Decision Tree | N/A | Decision Tree |
| **Time Horizon** | Not Stated. | N/A | Not stated. |
| **Discounting** | Not Stated. | N/A | Not stated. |
| **Sensitivity analysis** | Univariate | N/A | One-way. |
| **Key scenarios/variables in sensitivity analysis.** | Costs of tests and treatments. | N/A | Unit costs of DiaskinTest and TST. |
| **WTP Threshold** | Not Stated. | N/A | Not Stated. |
| **Unit Costs** | Breakdown of TST: Syringe 1.46 RUB, TST test and readout 26.14 RUB, one 0.2 ml dose of TST 1.5 RUB.  DiaskinTest: 256.71 | Strategy/Examination  TST: 107926 / 132  DiaskinTest: 52128 / 64 | Strategy:  DiaskinTest: 380.36 TST: 218.81 TST+ DiaskinTest: 220.46  Unit Cost of Test: DiaskinTest: 148 TST: 104.7 |

**Continuation of Table D**

| **Study Characteristics** | **Moiseeva 2014** | **Solodun 2017 (4)** | **Sinitsyn 2018** |
| --- | --- | --- | --- |
| **Country Setting** | Russia | Russia | Russia |
| **Year of Cost Valuation** | 2013 | Not stated. | 2015 |
| **Currency** | Rubles | Rubles | Rubles |
| **Study Population** | Children | Children and Adolescents | People living with HIV |
| **Index Diagnostic Test Strategies** | DiaskinTest   1. TST+ DiaskinTest | 1. DiaskinTest 2. TST + DiaskinTest | DiaskinTest |
| **Alternative Provided** | TST | TST | Do nothing. |
| **Type of economic evaluation** | CEA/CUA  Depends on effectiveness measure. | CEA | CBA |
| **Source of costing** | In accordance with the price list of GBUZ IC “KKPTD” as of 1.09.2013 | State Register and Moscow Centre for Tuberculosis. | Empirical Data Collection |
| **Primary outcome** | ICER: RUB/case of active TB | ICER: RUB/case of active TB identified. | Net savings |
| **Type of model** | Unclear | Decision Tree | N/A |
| **Time Horizon** | Not Stated. | Not stated. | Not Stated. |
| **Discounting** | Not Stated. | Not stated. | Not Stated. |
| **Sensitivity analysis** | None carried out. | One-way. | None carried out. |
| **Key scenarios/variables in sensitivity analysis.** | N/A | Cost of tests and treatment, sensitivity, and specificity of tests. | N/A |
| **WTP Threshold** | Not stated. | Not stated. | Not stated. |
| **Unit Costs** | TST: 85 74 RUB  DiaskinTest: 118.48 RUB | Strategy (RUB/100 diagnoses):  TST: 18,555.18  TST+ DiaskinTest: 16,311.93  DiaskinTest: 14,811.92  Unit Cost of Tests: DiaskinTest: 95.04 TST: 90.00 | Test/Strategy:  DiaskinTest: 720 RUB/2363.26 |

**Continuation of Table D**

| **Study Characteristics** | **Chugaev 2020 (5)** | **Steffen (2020)** (6) |
| --- | --- | --- |
| **Country Setting** | Russia | Brazil |
| **Year of Cost Valuation** | 2013 for TST | 2020 |
|  | 2019 for DiaskinTest |  |
| **Currency** | Rubles | USD |
| **Study Population** | Children | Adults living with HIV |
| **Index Diagnostic Test Strategies** | DiaskinTest | 1.  TST, 2.  QFT-GIT, e. EC test |
|  |  |  |
| **Alternative Provided** | TST | DiaskinTest |
| **Type of economic evaluation** | Cost analysis | CUA |
| **Source of costing** | Retrospective cohort study. | Ministry of Health and |
| **Primary outcome** | Cost of strategy and cost of additional diagnosis of TB. | ICER: $/QALY gained |
| **Type of model** | N/A | Markov. |
| **Time Horizon** | N/A | 20 years. |
| **Discounting** | N/A | 5% |
| **Sensitivity analysis** | None. | One-way, Two-way and PSA |
| **Key scenarios/variables in sensitivity analysis.** | Not stated. | TST and DiaskinTest sensitivity and specificity, prevalence LTBI |
| **WTP Threshold** | Not Stated. | $7544 |
| **Unit Costs** | No breakdown provided. | Yes |

# **III. Full data extraction of the results for all articles included in the secondary review**

**Table E:** Data extraction results for all the entries from the secondary systematic review

| **Study Characteristics** | **Linas (2011) (7)** | **Pareek (2011)(8)** | **del Campo (2012) (9)** |
| --- | --- | --- | --- |
| **Country Setting** | USA | UK | Spain |
| **Year of Cost Valuation** | 2011 | 2010 | 2012 |
| **Currency** | USD | GBP | Euros |
| **Study Population** | 1. Immigrant/Migrants 2. Immunocompromised 3. Vulnerable | Recently arrived immigrants (<16y.o. and 16- 35y.o.) | Healthcare workers. |
| **Index Diagnostic Test Strategies** | 1. QFT 2. TST | IGRA | 1. TST (10mm) 2. QFT 3. TST (5mm) + QFT |
| **Alternatives Provided** | No screening. | No screening | TST (5mm) |
| **Type of economic evaluation** | CUA | CEA | CEA |
| **Source of costing** | Published Literature | Empirical data collection | Published Literature (tests) and Empirical data collection for other costs. |
| **Primary outcome** | ICER: $/QALYs gained | ICER: £/active TB case averted. | ICER: Euro/active TB case averted. |
| **Type of model** | Markov. | Decision tree. | Decision Tree |
| **Time Horizon** | Lifetime. | 20 years. | 2 years. |
| **Discounting** | 1.5% | 3.5% | Not stated. |

| **Sensitivity analysis** | One-way, Two-way  deterministic. | One-way  deterministic. | One-way deterministic. |
| --- | --- | --- | --- |
| **Key scenarios/variables in**  **sensitivity analysis.** | TST and QFT sensitivity, specificity, and test costs. | Reactivation Rate, sensitivity, and specificity of tests. | TST and QFT sensitivity, specificity and LTBI prevalence. |
| **WTP Threshold** | $100,000 | Not Stated. | Not Stated. |

**Continuation of Table E**

| **Study Characteristics** | **Eralp. (2012) (10)** | **Shah (2012) (11)** | **Mandalakas (2013) (12)** |
| --- | --- | --- | --- |
| **Country Setting** | UK | USA | South Africa |
| **Year of Cost Valuation** | 2011 | 2012 | 2009 |
| **Currency** | GBP | USD | USD |
| **Study Population** | Healthcare Workers | Individuals at primary health clinic with positive TST. | Children (0-2 and 3-5y.o.) contacts. |
| **Index Diagnostic Test Strategies** | 1. QFT 2. TST | TST + QFT-GIT | 1) TST 2) TST+QFT.  3) TST-QFT. 4) TST+ T-SPOT  5) TST- T-SPOT 6) QFT.  7) T-SPOT |
| **Alternative Provided** | TST + QFT | TST | No Screening |
| **Type of economic**  **evaluation** | CEA | CEA | CEA |
| **Source of costing** | Published Literature | Data from Boston  Health Dep. | Published Literature. |
| **Primary outcome** | ICER: £/Life Year Gained. | ICER: $/QALY gained. | ICER: $/active TB case averted. |
| **Type of model** | Markov. | Decision tree. | Markov |
| **Time Horizon** | 20 years. | 1 year for those without LTBI and lifetime for those with  LTBI. | 15 years. |
| **Discounting** | 5% | 3% | 3% |
| **Sensitivity analysis** | One-way deterministic  and PSA. | Two-way and PSA. | One-way deterministic. |
| **Key scenarios/variables in**  **sensitivity analysis.** | TST and QFT sensitivity, specificity, test costs  and LTBI prevalence. | QFT-GIT sensitivity, specificity and LTBI  prevalence. | TST and QFT sensitivity, specificity and LTBI prevalence. |
| **WTP Threshold** | £30,000 | $50,000 | Not Stated. |

**Continuation of Table E**

| **Study Characteristics** | **Pareek (2013) (13)** | **Steffen (2013) (14)** | **Swaminath (2013) (15)** |
| --- | --- | --- | --- |
| **Country Setting** | UK | USA | USA |
| **Year of Cost Valuation** | 2011 | 2012 | Not stated. |
| **Currency** | GBP | USD | USD |
| **Study Population** | Recently arrived  immigrants (≤35 y.o.). | 35y.o. close contacts. | Immunosuppressed with IBD |
| **Index Diagnostic Test Strategies** | 1. T-SPOT 2. QFT 3. TST + T-SPOT 4. TST + QFT | 1. QFT 2. TST + QFT | QFT |
| **Alternative Provided** | TST | TST | TST |
| **Type of economic**  **evaluation** | CEA | CEA | CEA |
| **Source of costing** | Published Literature | Published Literature. | Published Literature. |
| **Primary outcome** | ICER: £/active TB case averted. | ICER: $/active TB case  averted. | Cost and TB deaths. |
| **Type of model** | Decision Tree | Decision tree. | Decision Tree |
| **Time Horizon** | 20 years. | 2 years. | 1 year. |
| **Discounting** | 3.5% | None. | None. |
| **Sensitivity analysis** | One-way  deterministic. | One-way and two-way  and PSA. | One-way deterministic. |
| **Key scenarios/variables in sensitivity analysis.** | TST and QFT sensitivity, specificity, test costs and LTBI  prevalence. | TST and QFT sensitivity, specificity, test costs and LTBI prevalence. | TST and QFT sensitivity, specificity, test, and treatment. costs and LTBI  prevalence. |
| **WTP Threshold** | Not Stated. | $50,000 | Not Stated. |

**Continuation of Table E**

| **Study Characteristics** | **Verma (2013) (16)** | **Capocci, (2015) (17)** | **Wingate (2015) (18)** |
| --- | --- | --- | --- |
| **Country Setting** | Canada | UK | USA |
| **Year of Cost Valuation** | 2012 | 2012 | 2012 |
| **Currency** | Canadian Dollars | Euro | USD |
| **Study Population** | >65y.o. in long term  care | Adults living with HIV | Pre arrival refugees  to USA. |
| **Index Diagnostic Test Strategies** | TST | 1. QFT + TST 2. QFT (for higher risk) 3. QFT (for all) | TST |
| **Alternative Provided** | No screening | No Screening | No Screening |
| **Type of economic**  **evaluation** | CEA | CEA/CUA | CBA |
| **Source of costing** | Published Literature | Published Literature and clinic data. | Published Literature and experts. |
| **Primary outcome** | ICER: $/active TB case averted. | ICER: Euro/QALY gained or active TB case averted. | Net benefit (Cost) |
| **Type of model** | Markov | Markov. | Decision tree and  Markov. |
| **Time Horizon** | 4 years | Lifetime | 20 years. |
| **Discounting** | 3% | 3.5% | 3% |
| **Sensitivity analysis** | One-way  deterministic. | PSA. | One-way  deterministic. |
| **Key scenarios/variables in sensitivity analysis.** | TST and QFT sensitivity, specificity and LTBI prevalence. | TST and QFT sensitivity, specificity, test, and treatment. costs and LTBI prevalence. | TST and QFT sensitivity, specificity, test costs and LTBI prevalence. |
| **WTP Threshold** | Not Stated. | Euro 24,000 | Not Stated. |

**Continuation of Table E**

| **Study Characteristics** | **Auguste. (2016)(19)** | **Nijhawan (2016) (20)** | **Campbell (2017) (21)** |
| --- | --- | --- | --- |
| **Country Setting** | UK | USA | Canada |
| **Year of Cost Valuation** | 2012 | 2013 | 2016 |
| **Currency** | Euro | USD | Canadian Dollars |
| **Study Population** | 1. Children 2. Immunocompromised 3. Recently Arrived Immigrants 4. General Population. | Adults entering jail. | Pre-arrival refugees to USA. |
| **Index Diagnostic Test Strategies** | 1. IGRA 2. TST + IGRA 3. Simultaneous. | QFT | 1. TST 2. IGRA 3. TST + IGRA |
| **Alternative Provided** | TST | TST | No Screening |
| **Type of economic**  **evaluation** | CUA | Cost Analysis. | CUA |
| **Source of costing** | Published Literature, NHS data and assumptions. | Published Literature  and empirical data collection. | British Columbia  Centre for Disease control |
| **Primary outcome** | ICER: £/QALY gained | Cost difference per active TB case  detected. | ICER: $/QALY  gained. |
| **Type of model** | Decision tree and discrete  event simulation. | Decision tree. | Discrete event  simulation. |
| **Time Horizon** | Lifetime | Not Stated. | 10 years. |
| **Discounting** | 3.5% | Not Stated. | 1.5% |
| **Sensitivity analysis** | PSA. | One-way. | One-way and  PSA. |
| **Key scenarios/variables in sensitivity analysis.** | TST and QFT sensitivity, specificity, test, and treatment. costs and LTBI prevalence. | Cost of labour, unit cost of tests. | TST and QFT sensitivity, specificity, test costs and LTBI  prevalence. |
| **WTP Threshold** | £30,000 | Not Stated. | $100,000 |

**Continuation of Table E**

| **Study Characteristics** | **Haukaas (2017) (22)** | **Mullie (2017) (23)** | **Tasillo, (2017) (24)** |
| --- | --- | --- | --- |
| **Country Setting** | Norway | Canada | USA |
| **Year of Cost Valuation** | 2013 | 2015 | 2015 |
| **Currency** | Euro | Canadian Dollars | USD |
| **Study Population** | Recently arrived immigrants <35y.o. | HCW with negative TST at time of  employment. | US-born or migrants living with or without  comorbidities. |
| **Index Diagnostic Test Strategies** | 1. QFT (for those with risk factors) 2. TST + QFT 3. QFT (for all) | QFT-GIT | 1. TST 2. IGRA 3. IGRA + TST 4. IGRA - TST |
| **Alternative Provided** | No Screening | TST | No Screening |
| **Type of economic**  **evaluation** | CEA | CUA | CUA |
| **Source of costing** | Published Literature  and expert opinion. | Published  Literature. | Assumptions and  published literature. |
| **Primary outcome** | ICER: £/QALY gained | ICER: $/QALY  gained. | ICER: $/QALY gained. |
| **Type of model** | Decision Tree and  Markov | Decision tree. | Decision tree and  Markov. |
| **Time Horizon** | 10 years. | 20 years. | Lifetime |
| **Discounting** | 4% | 3% | 3% |
| **Sensitivity analysis** | One-way. | One-way and two  scenario analyses. | One-way and PSA. |
| **Key**  **scenarios/variables in sensitivity analysis.** | TST and QFT sensitivity,  specificity, test, costs and LTBI prevalence. | TST and QFT  sensitivities and specificities. | TST and QFT sensitivity,  specificity, test costs and LTBI prevalence. |
| **WTP Threshold** | 28,400 Euros | Not Stated. | $100,000 |

**Continuation of Table E**

| **Study Characteristics** | **Abubakar (2018) (25)** | **Li (2018) (26)** | **Sohn (2018) (27)** |
| --- | --- | --- | --- |
| **Country Setting** | UK | Hong Kong | Japan |
| **Year of Cost**  **Valuation** | Not Stated. | Not stated. | 2015 |
| **Currency** | GBP | USD | USD |
| **Study Population** | 1. Recent immigrants. 2. Contacts | Elderly (>65y.o.) at admission to residential care  home. | Adolescents (13- 18y.o.) contacts. |
| **Index Diagnostic Test Strategies** | 1. T-SPOT.TB 2. QFT-GIT  3. TST (varying cut offs)  4. Confirm positive or negative T-SPOT or QFT-GIT after TST | IGRA | 1. TST + QFT 2. QFT |
| **Alternative Provided** | No Screening | No screening. | TST |
| **Type of economic**  **evaluation** | CUA | CUA | CEA |
| **Source of costing** | Published literature and  NHS Data. | Estimation. | Published literature. |
| **Primary outcome** | ICER: £/QALY gained. | ICER: $/QALY  gained. | ICER: $/QALY gained. |
| **Type of model** | Decision Tree. | Markov. | Decision tree. |
| **Time Horizon** | Lifetime | 20 years. | 2 years. |
| **Discounting** | 3.5% | 5% | 3% (overhead costs  only) |
| **Sensitivity analysis** | PSA. | One-way and PSA. | One-way and PSA. |
| **Key scenarios/variables in sensitivity**  **analysis.** | Test sensitivity and specificity. | TST and QFT sensitivities and specificities,  reactivation rate. | TST and QFT sensitivity, specificity, test costs and LTBI  prevalence. |
| **WTP Threshold** | £20,000 | $50,000 | $50,000 |

**Continuation of Table E**

| **Study Characteristics** | **Campbell (2019a) (28)** | **Campbell (2019b) (29)** | **Loureiro (2019) (30)** |
| --- | --- | --- | --- |
| **Country Setting** | Canada | Canada | Brazil |
| **Year of Cost**  **Valuation** | 2016 | 2016 | 2016 |
| **Currency** | Canadian Dollars | Canadian Dollars | USD |
| **Study Population** | Pre-arrival immigrants. | Migrants with  either late-stage CKD or beginning  dialysis. | Primary HCW. |
| **Index Diagnostic Test Strategies** | 1. QFT-GIT 2. TST + QFT-GIT 3. TST (>10mm) | 1. QFT-GIT 2. TST (>10mm) | 1. TST (>10mm) 2. TST (>10mm) + QFT 3. TST (>5mm) + QFT QFT |
| **Alternative Provided (Baseline for Incremental**  **Analysis)** | No screening | No screening | TST (>5mm) |
| **Type of economic**  **evaluation** | CUA | CUA | CEA |
| **Source of costing** | British Columbia Centre for Disease control and expert opinion. | British Columbia Centre for Disease control and expert  opinion. | Ministry of Health and estimations. |
| **Primary outcome** | ICER: $/QALY gained | ICER: $/QALY  gained. | ICER: $/active TB case  averted. |
| **Type of model** | Discrete event  simulation | Markov. | Decision tree. |
| **Time Horizon** | 25 years. | 25 years. | 1 year. |
| **Discounting** | 3% | 3% | None. |
| **Sensitivity analysis** | PSA. | PSA. | One-way. |
| **Key scenarios/variables in sensitivity analysis.** | TST and QFT sensitivity and specificity, incidence rate of country of origin of immigrant, reactivation  rate. | TST and QFT sensitivities and specificities, cost of tests and reactivation rate. | TST and QFT sensitivity, specificity, test costs and LTBI prevalence. |
| **WTP Threshold** | $50,000 | $50,000 | Not stated. |

**Continuation of Table E**

| **Study Characteristics** | **Png (2019) (31)** | **Al Abri (2020) (32)** | **Jo (2020) (33)** |
| --- | --- | --- | --- |
| **Country Setting** | Singapore | Oman | USA |
| **Year of Cost**  **Valuation** | 2016 | 2020 | 2018 |
| **Currency** | USD | USD | USD |
| **Study Population** | HCW. | 20-year-old recent immigrants. | 1. non–US-born, 2. living with diabetes, 3. HIV-positive, 4. experiencing recent homelessness, 5. incarcerated |
| **Index Diagnostic Test Strategies** | QFT-GIT either annually or every three years for combination of 1) new hires 2) high risk 3)  international 4)  universal. | 1. QFT 2. TST 3. CXR   All with varying treatments. | IGRA |
| **Alternative Provided (Baseline for Incremental**  **Analysis)** | No Screening | QFT (4-month RIF) | Not Stated. |
| **Type of economic**  **evaluation** | CUA | CUA | CUA |
| **Source of costing** | National University  Hospital and Published Literature. | Not Stated. | Published Literature. |
| **Primary outcome** | ICER: $/QALY gained | ICER: $/QALY  gained | ICER: $/QALY gained. |
| **Type of model** | Decision Tree. | Markov. | Individual-based TB  epidemiological model. |
| **Time Horizon** | 3 years. | Lifetime | 30 years. |
| **Discounting** | 3% | Not stated. | 3% |
| **Sensitivity analysis** | PSA. | One-way, Two-way  and PSA. | One-way and PSA. |
| **Key scenarios/variables in sensitivity analysis.** | TST and QFT sensitivity and specificity, incidence rate of country of origin of immigrant, reactivation rate. | TST and QFT sensitivity and specificity, incidence rate of country of origin of immigrant,  reactivation rate. | Cost of tests, treatment, and completion of treatment probability. |
| **WTP Threshold** | $50,000 | $100,000 | Not stated. |

**Continuation of Table E**

| **Study Characteristics** | **Steffen (2020) (6)** | **Kim (2018) (34)** |
| --- | --- | --- |
| **Country Setting** | Brazil | South Africa |
| **Year of Cost**  **Valuation** | 2020 | 2016 |
| **Currency** | USD | USD |
| **Study Population** | Adults living with HIV | HIV+ pregnant women |
| **Index Diagnostic Test Strategies** | 1. TST 2. QFT-GIT 3. EC Test | 1. TST |
| **Alternative Provided**  **(Baseline for Incremental Analysis)** | DiaskinTest | QFT-GIT |
| **Type of economic**  **evaluation** | CUA | CEA |
| **Source of costing** | Ministry of Health and  market value. | National Health Laboratory service |
| **Primary outcome** | ICER: $/QALY gained | ICER: $/DALY averted |
| **Type of model** | Markov. | Decision tree |
| **Time Horizon** | 20 years. | 12 months |
| **Discounting** | 5% | 3% |
| **Sensitivity analysis** | One-way, Two-way and  PSA. | One-way, Two-way and  PSA. |
| **Key scenarios/variables in sensitivity analysis.** | TST and DiaskinTest sensitivity and specificity, prevalence  of LTBI. | TST and QFT sensitivity and specificity, and other highly sensible parameters |
| **WTP Threshold** | $7544 | $12,860/DALY |

**Continuation of Table E**

| **Study Characteristics** | **Auguste (2022) (35)** | **Fekadu (2022) (36)** | **Marx (2021) (37)** |
| --- | --- | --- | --- |
| **Country Setting** | UK | USA | Germany |
| **Year of Cost**  **Valuation** | 2020 | 2021 | 2020 |
| **Currency** | GBP | USD | Euro |
| **Study Population** | Adults living with HIV | Adults living with HIV | Asylum seekers |
| **Index Diagnostic Test Strategies** | 1.TST   1. QFT-GIT 2. T-SPOT.TB 3. QFT-GIT and TST5mm | No testing strategy, three single-test strategies (TST; T-SPOT.TB; QFT-Plus), four sequential strategies to confirm negative tests (TST-negative followed by T-SPOT.TB; TST-negative followed by QFT-Plus; T-SPOT.TB-negative followed by TST and QFT-Plus-negative followed by TST) and four sequential strategies to confirm positive tests (TST-positive followed by T-SPOT.TB, TST-positive followed by QFT-Plus, T-SPOT. TB-positive followed by TST and QFT-Plus-positive followed by TST), and literature. | 1. QFT-GIT |
| **Alternative Provided**  **(Baseline for Incremental Analysis)** | No-testing scenario | Less costly option | No LTBI screening, no TPT |
| **Type of economic**  **evaluation** | CEA | CEA | CEA |
| **Source of costing** | UK National Health Service (NHS) and literature. | Centres for Medicare and Medicaid Services clinical laboratory fee schedule |  |
| **Primary outcome** | ICER: $/QALY gained | ICER: $/QALY gained | ICER: $/QALY gained |
| **Type of model** | Decision tree + Markov model | Decision tree + Markov model | Decision tree + Markov model |
| **Time Horizon** | - | 20 years | - |
| **Discounting** | 3.5% | 3% | 3% |
| **Sensitivity analysis** | One-way, Two-way and  PSA. | One-way PSA. | One-way PSA. |
| **Key scenarios/variables in sensitivity analysis.** | Specificity of tests and prevalence of latent tuberculosis infection | TBI prevalence in foreign-born HIV patients, TB disease treatment success rate, TBI prevalence in US-born HIV patients, proportion of foreign-born among HIV patients, and TB risk reduction rate after completed TBI treatment. | The probability of LTBI reactivation, the effectiveness of TPT, the TB case-fatality ratio, the specificity of IGRA, and the cost for LTBI screening |
| **WTP Threshold** | $27074/QALY | $200000/QALY | $91447/QALY |

# **IV. Assessing studies quality using Drummond’s checklist for novel skin test**

**Table F.** Drummond checklist for studies quality: Cost/Cost-effectiveness analyses for Novel skin tests for diagnosing TBI

| **Drummond Checklist Questions** | **Kulikov 2009** | **Aksenova 2011** | **Yagudina 2013** |
| --- | --- | --- | --- |
| **1.     Was a well-defined question posed in answerable form?** |  |  |  |
| 1.1. Did the study examine both costs and effects of the service(s) or programme(s)? | Yes | No, only presents costs | Yes |
| 1.2. Did the study involve a comparison of alternatives? | Yes | Yes | Yes |
| 1.3. Was a viewpoint for the analysis stated and was the study placed in any decision-making context? | Yes, children and adolescents | Yes, children | Yes |
| **2. Was a comprehensive description of the competing alternatives given?** |  |  |  |
| 2.1. Were there any important alternatives omitted? | No | No | No |
| 2.2. Was (should) a do-nothing alternative be considered? | No | No | No |
| **3.    Was the effectiveness of the programme or services established?** |  |  |  |
| 3.1. Was this done through a randomised, controlled clinical trial? If so, did the trial protocol reflect what would happen in regular practice? | No | No | No |
| 3.2. Was effectiveness established through an overview of clinical studies? | Yes.  Sensitivity and specificity of tests values from number of clinical studies. | No, cost analysis, no CE | Yes |
| 3.3. Were observational data or assumptions used to establish effectiveness? If so, what are the potential biases in results? | Yes. Observational data, no time horizon/discounting provided. | N/A | Assumption on the proportion of patients with a dubious and positive test with 2TE PPD-L and Diaskintest drug® to be equal to two different existing artiles, which might lead to biased conclusion depending on the settings analysed and population characteristics |
| **4. Were all the important and relevant costs and consequences for each alternative identified?** |  |  |  |
| 4.1. Was the range wide enough for the research question at hand? | Yes | Yes | Yes |
| 4.2. Did it cover all relevant viewpoints? | Yes | Yes | Yes |
| 4.3. Were the capital costs, as well as operating costs, included? | Test and treatment costs included, the rest is unclear | Only research costs, sample analyses costs, preventive treatment, consultation and Xrays | Yes, it included treatment, diagnostic, drugs and chemoteraphy costs, registering and operating costs, among others |
| **5.    Were costs and consequences measured accurately in appropriate physical units?** |  |  |  |
| 5.1. Were any of the identified items omitted from measurement? If so, does this mean that they carried no weight in the subsequent analysis? | Outcome costs is missing | The cost of test itself, it just measures the cost of diagnostic measures. And outcome costs | No |
| 5.2. Were there any special circumstances (e.g., joint use of resources) that made measurement difficult? Were these circumstances handled appropriately? | No | No | No |
| **6.    Were the cost and consequences valued credibly?** |  |  |  |
| 6.1. Were the sources of all values clearly identified? | Unclear, cost of valuation is not stated | Unclear, but only stated on the Table 3 | Yes |
| 6.2. Were market values employed for changes involving resources gained or depleted? | Yes | Yes | Yes |
| 6.3. Where market values were absent (e.g. volunteer labour), or market values did not reflect actual values (such as clinic space donated at a reduced rate), were adjustments made to approximate market values? | N/A | N/A | N/A |
| 6.4. Was the valuation of consequences appropriate for the question posed? | Yes | N/A | Yes |
| **7.    Were costs and consequences adjusted for differential timing?** |  |  |  |
| 7.1. Were costs and consequences that occur in the future ‘discounted’ to their present values? | No | N/A | Not stated |
| 7.2. Was there any justification given for the discount rate used? | Discounting rate No stated | N/A | N/A |
| **8.    Was an incremental analysis of costs and consequences of alternatives performed?** |  |  |  |
| 8.1. Were the additional (incremental) costs generated by one alternative over another compared to the additional effects, benefits, or utilities generated? | Yes | No | Yes |
| **9.    Was allowance made for uncertainty in the estimates of costs and consequences?** |  |  |  |
| 9.1. If data on costs and consequences were stochastic (randomly determined sequence of observations), were appropriate statistical analyses performed? | Yes | N/A | Yes |
| 9.2. If sensitivity analysis was employed, was justification provided for the range of values (or for key study parameters)? | Yes, for treatment and test costs | N/A | No explanation, just stated the parameters over which the SA was employed (test cost) |
| 9.3. Were the study results sensitive to changes in the values? | Not that much. The conclusion was robust to changes in key parameters with the cost of a second clinical visit being the most influential to the cost-effectiveness ratios | N/A | No, it was stable |
| **10.    Did the presentation and discussion of study results include all issues of concern to users?** |  |  |  |
| 10.1. Were the conclusions of the analysis based on some overall index or ratio of costs to consequences (e.g. cost-effectiveness ratio)? | Yes (2.28 rubles compared to 3.42) |  | Yes |
| 10.2. Were the results compared with those of others who have investigated the same question? If so, were allowances made for potential differences in study methodology? | No | No | No |
| 10.3. Did the study discuss the generalisability of the results to other settings and patient/client groups? | No | Yes | No |
| 10.4. Did the study allude to, or take account of, other important factors in the choice or decision under consideration (e.g. distribution of costs and consequences, or relevant ethical issues)? | No | Yes | Yes |
| 10.5. Did the study discuss issues of implementation, such as the feasibility of adopting the ‘preferred’ programme given existing financial or other constraints, and whether any freed resources could be redeployed to other worthwhile programmes? | Yes | Yes | No |

Continuation of **Table F**

| **Drummond Checklist Questions** | **Moiseeva 2014** | **Solodun 2017** | **Sinitsyn 2018** | **Chugaev 2020** |
| --- | --- | --- | --- | --- |
| **1.     Was a well-defined question posed in answerable form?** |  |  |  |  |
| 1.1. Did the study examine both costs and effects of the service(s) or programme(s)? | Yes | Yes | Yes | Yes |
| 1.2. Did the study involve a comparison of alternatives? | Yes | Yes | Yes | Yes |
| 1.3. Was a viewpoint for the analysis stated and was the study placed in any decision-making context? | Yes | Yes, children and adolescents | Yes, HIV patients | Yes, children |
| **2. Was a comprehensive description of the competing alternatives given?** |  |  |  |  |
| 2.1. Were there any important alternatives omitted? | No | No | Yes, the use of any other test rather than Diaskin solely | Yes |
| 2.2. Was (should) a do-nothing alternative be considered? | No | No | Yes | No |
| **3.    Was the effectiveness of the programme or services established?** |  |  |  |  |
| 3.1. Was this done through a randomised, controlled clinical trial? If so, did the trial protocol reflect what would happen in regular practice? | No | No | No | No |
| 3.2. Was effectiveness established through an overview of clinical studies? | Yes | Yes | Yes, CB analysis | Yes |
| 3.3. Were observational data or assumptions used to establish effectiveness? If so, what are the potential biases in results? | Not clear CE method used | Yes, it uses observational data from specific settings | Yes, ICER is not computed and no other interventions were employed rather than do-nothing and Diaskintest |  |
| **4. Were all the important and relevant costs and consequences for each alternative identified?** |  |  |  |  |
| 4.1. Was the range wide enough for the research question at hand? | Yes | Yes | Yes | Yes |
| 4.2. Did it cover all relevant viewpoints? | Yes | Yes | Yes | Yes |
| 4.3. Were the capital costs, as well as operating costs, included? | unclear | Yes, including staff costs, diagnostics, tests, operating costs, etc | No | No |
| **5.    Were costs and consequences measured accurately in appropriate physical units?** |  |  |  |  |
| 5.1. Were any of the identified items omitted from measurement? If so, does this mean that they carried no weight in the subsequent analysis? | No | No | No | No |
| 5.2. Were there any special circumstances (e.g., joint use of resources) that made measurement difficult? Were these circumstances handled appropriately? | No | No | No | No |
| **6.    Were the cost and consequences valued credibly?** |  |  |  |  |
| 6.1. Were the sources of all values clearly identified? | Unclear | Yes, most costs coming from the State Treasure Healthcare institution | Yes |  |
| 6.2. Were market values employed for changes involving resources gained or depleted? | Yes | Yes | Yes | Yes |
| 6.3. Where market values were absent (e.g. volunteer labour), or market values did not reflect actual values (such as clinic space donated at a reduced rate), were adjustments made to approximate market values? | N/A | N/A | N/A | N/A |
| 6.4. Was the valuation of consequences appropriate for the question posed? | Yes | Yes | Yes | Yes |
| **7.    Were costs and consequences adjusted for differential timing?** |  |  |  |  |
| 7.1. Were costs and consequences that occur in the future ‘discounted’ to their present values? | No | Not stated | Not stated | No |
| 7.2. Was there any justification given for the discount rate used? | N/A | N/A | N/A | N/A |
| **8.    Was an incremental analysis of costs and consequences of alternatives performed?** |  |  |  |  |
| 8.1. Were the additional (incremental) costs generated by one alternative over another compared to the additional effects, benefits, or utilities generated? | Yes | Yes | Non ICER computed, comparison between two alternatives | Yes Diaskin over TST, costs saved |
| **9.    Was allowance made for uncertainty in the estimates of costs and consequences?** |  |  |  |  |
| 9.1. If data on costs and consequences were stochastic (randomly determined sequence of observations), were appropriate statistical analyses performed? | No | N/A | N/A | N/A |
| 9.2. If sensitivity analysis was employed, was justification provided for the range of values (or for key study parameters)? | No sensitivity analyses carried out | Yes | Non sensitivity analyses employed | Non sensitivity analyses employed |
| 9.3. Were the study results sensitive to changes in the values? | N/A | No, 1% change | N/A | N/A |
| **10.    Did the presentation and discussion of study results include all issues of concern to users?** |  |  |  |  |
| 10.1. Were the conclusions of the analysis based on some overall index or ratio of costs to consequences (e.g. cost-effectiveness ratio)? | No | Not stated | No | No |
| 10.2. Were the results compared with those of others who have investigated the same question? If so, were allowances made for potential differences in study methodology? | No | No | No | No |
| 10.3. Did the study discuss the generalisability of the results to other settings and patient/client groups? | No | No | No | Yes |
| 10.4. Did the study allude to, or take account of, other important factors in the choice or decision under consideration (e.g. distribution of costs and consequences, or relevant ethical issues)? | No | No | No | No |
| 10.5. Did the study discuss issues of implementation, such as the feasibility of adopting the ‘preferred’ programme given existing financial or other constraints, and whether any freed resources could be redeployed to other worthwhile programmes? | No | No | No | Yes |

# **V. Assessing studies quality using Drummond’s checklist for TST or IGRA tests**

**Table G.** Drummond Checklist for studies quality: Cost/Cost-effectiveness analyses for TST or IGRA for diagnosing TBI

| **Drummond Checklist Questions** | **Linas (2011)** | **Pareek (2011)** | **del Campo (2012)** | **Eralp (2012)** | **Shah (2012)** |
| --- | --- | --- | --- | --- | --- |
| 1. **Was a well-defined question posed in answerable form?** | | | | | |
| 1.1. Did the study examine both costs and effects of the service(s) or programme(s)? | Yes | Yes | Yes | Yes | Yes |
| 1.2. Did the study involve a comparison of alternatives? | Yes | Yes | Yes | Yes | Yes |
| 1.3. Was a viewpoint for the analysis stated and was the study placed in any decision-making context? | Yes  Immigrant/migrants, immunocompromised and vulnerable populations in the USA. | Yes  Recently arrived immigrants  (<16y.o. and 16-35y.o.) in the UK. | Yes  Healthcare workers in Spain. | Yes  Healthcare workers in the UK. | Yes  Individuals at primary health clinic with positive TST in the USA. |
| **2. Was a comprehensive description of the competing alternatives given?** | | | | | |
| 2.1. Were there any important alternatives omitted? | No | No | No | No | No |
| 2.2. Was (should) a do-nothing alternative be considered? | Yes, do nothing was included appropriately. | Yes, do nothing was included appropriately. | No | No | No |
| **3.    Was the effectiveness of the programme or services established?** | | | | | |
| 3.1. Was this done through a randomised, controlled clinical trial? If so, did the trial protocol reflect what would happen in regular practice? | No | No | No | No | No |
| 3.2. Was effectiveness established through an overview of clinical studies? | Yes.  Sensitivity and specificity of tests values from number of clinical studies. | Yes.  Sensitivity and specificity of tests values from number of clinical studies. | Yes.  Sensitivity and specificity of tests values from number of clinical studies. | Yes.  Sensitivity and specificity of tests values from number of clinical studies. | Yes.  Sensitivity and specificity of tests values from number of clinical studies. |
| 3.3. Were observational data or assumptions used to establish effectiveness? If so, what are the potential biases in results? | Yes.  Assumed that quality of life with cured TB was the same as that for healthy individuals.  All assumptions references/reasoned adequately. | Yes.  Prospective cohort analysis performed for LTBI prevalence.  All assumptions references/reasoned adequately. | Yes.  Key assumption that no active cases of TB at the time of testing.  All assumptions references/reasoned adequately. | Yes  Key assumption that “LTBI generates a positive result at same probability that active TB” for test.  All effectiveness assumptions clearly identified and references/ reasoned adequately. | Yes  All effectiveness assumptions clearly identified and references/ reasoned adequately. |
| **4. Were all the important and relevant costs and consequences for each alternative identified?** | | | | | |
| 4.1. Was the range wide enough for the research question at hand? | Yes | Yes | Yes | Yes | Yes |
| 4.2. Did it cover all relevant viewpoints? | Yes | Yes | Yes | Yes | Yes |
| 4.3. Were the capital costs, as well as operating costs, included? | No.  State only direct medical costs included. | No. | No. | Yes, stated overheads included but no description provided. | Yes, operating costs such as quality assurance, specimen transport, supply delivery, and estimates for rent and utilities included. |
| **5.    Were costs and consequences measured accurately in appropriate physical units?** | | | | | |
| 5.1. Were any of the identified items omitted from measurement? If so, does this mean that they carried no weight in the subsequent analysis? | No | Yes.  Excluded drug-resistant strains and HIV infection.  Assumed minimal impact. | No | No | No |
| 5.2. Were there any special circumstances (e.g., joint use of resources) that made measurement difficult? Were these circumstances handled appropriately? | No | No | No | No | No |
| **6.    Were the cost and consequences valued credibly?** | | | | | |
| 6.1. Were the sources of all values clearly identified? | Yes | Yes | Yes | Yes | Yes |
| 6.2. Were market values employed for changes involving resources gained or depleted? | Yes | Yes | Yes | Yes | Yes |
| 6.3. Where market values were absent (e.g. volunteer labour), or market values did not reflect actual values (such as clinic space donated at a reduced rate), were adjustments made to approximate market values? | N/A | N/A | N/A | N/A | N/A |
| 6.4. Was the valuation of consequences appropriate for the question posed? | Yes | Yes | Yes | Yes | Yes |
| **7.    Were costs and consequences adjusted for differential timing?** | | | | | |
| 7.1. Were costs and consequences that occur in the future ‘discounted’ to their present values? | Yes, 3%. | Yes, 3.5%. | No. | Yes, 5%. | Yes, 3%. |
| 7.2. Was there any justification given for the discount rate used? | Yes, following Siegel et al. 1997 guidelines. | Yes, following NICE recommendations. | N/A | State “standard rate” with no reference in supplementary material. | No justification for discount rate of costs provided. |
| **8.    Was an incremental analysis of costs and consequences of alternatives performed?** | | | | | |
| 8.1. Were the additional (incremental) costs generated by one alternative over another compared to the additional effects, benefits, or utilities generated? | Yes | Yes | Yes | Yes | Yes |
| **9.    Was allowance made for uncertainty in the estimates of costs and consequences?** | | | | | |
| 9.1. If data on costs and consequences were stochastic (randomly determined sequence of observations), were appropriate statistical analyses performed? | Yes | Yes | Yes | Yes | Yes |
| 9.2. If sensitivity analysis was employed, was justification provided for the range of values (or for key study parameters)? | Yes | Yes | Yes | Yes | Yes |
| 9.3. Were the study results sensitive to changes in the values? | Yes – many conclusions for different populations sensitive to key parameters. | No | No | No | No |
| **10.    Did the presentation and discussion of study results include all issues of concern to users?** | | | | | |
| 10.1. Were the conclusions of the analysis based on some overall index or ratio of costs to consequences (e.g. cost-effectiveness ratio)? | Yes | Yes | Yes | Yes | Yes |
| 10.2. Were the results compared with those of others who have investigated the same question? If so, were allowances made for potential differences in study methodology? | No | Yes | Yes | No | No |
| 10.3. Did the study discuss the generalisability of the results to other settings and patient/client groups? | No | Yes | No | No | Yes |
| 10.4. Did the study allude to, or take account of, other important factors in the choice or decision under consideration (e.g. distribution of costs and consequences, or relevant ethical issues)? | No | Yes | No | Yes | No |
| 10.5. Did the study discuss issues of implementation, such as the feasibility of adopting the ‘preferred’ programme given existing financial or other constraints, and whether any freed resources could be redeployed to other worthwhile programmes? | No | Yes | No | Yes | Yes |

Continuation of **Table G**

| **Drummond Checklist Questions** | **Mandalakas (2013)** | **Pareek (2013)** | **Steffen (2013)** | **Swaminath (2013)** | **Verma (2013)** |
| --- | --- | --- | --- | --- | --- |
| 1. **Was a well-defined question posed in answerable form?** | | | | | |
| 1.1. Did the study examine both costs and effects of the service(s) or programme(s)? | Yes | Yes | Yes | Yes | Yes |
| 1.2. Did the study involve a comparison of alternatives? | Yes | Yes | Yes | Yes | Yes |
| 1.3. Was a viewpoint for the analysis stated and was the study placed in any decision-making context? | Yes  Children (0-2 and 3-5y.o.) contacts in South Africa. | Yes  Recently arrived immigrants (≤35 y.o.) in the UK. | Yes  35y.o. close contacts of active TB cases in Brazil. | Yes  Immunosuppressed with IBD in the USA. | Yes  >65y.o. in long term care in Canada. |
| **2. Was a comprehensive description of the competing alternatives given?** | | | | | |
| 2.1. Were there any important alternatives omitted? | No | No | No | No | No |
| 2.2. Was (should) a do-nothing alternative be considered? | Yes, do nothing was included appropriately. | No | No | No | Yes, do nothing was included appropriately. |
| **3.    Was the effectiveness of the programme or services established?** | | | | | |
| 3.1. Was this done through a randomised, controlled clinical trial? If so, did the trial protocol reflect what would happen in regular practice? | No | No | No | No | No |
| 3.2. Was effectiveness established through an overview of clinical studies? | Yes.  Sensitivity and specificity of tests values from number of clinical studies. | Yes.  Sensitivity and specificity of tests values from number of clinical studies. | Yes.  Sensitivity and specificity of tests values from number of clinical studies. | Yes.  Sensitivity and specificity of tests values from number of clinical studies. | Yes  Sensitivity and specificity of tests values from number of clinical studies. |
| 3.3. Were observational data or assumptions used to establish effectiveness? If so, what are the potential biases in results? | Yes  All effectiveness assumptions are said to be derived from published data obtained in high-burden settings, but no reference provided. | Yes  Observational study performed to estimate LTBI prevalence. | Yes  Many assumptions effecting effectiveness with no references/reasoning. | Yes  Many assumptions to estimate effectiveness with only a small number with references/reasoning. | Yes  Key assumption is TST characteristics used are for general population despite evidence of lower specificity and sensitivity for elderly.  Could introduce overestimation of effectiveness hence underestimate of cost-effectiveness ratio. |
| **4. Were all the important and relevant costs and consequences for each alternative identified?** | | | | | |
| 4.1. Was the range wide enough for the research question at hand? | Yes | Yes | Yes | Yes | Yes |
| 4.2. Did it cover all relevant viewpoints? | Yes | Yes | Yes | Yes | Yes |
| 4.3. Were the capital costs, as well as operating costs, included? | Unclear.  Outpatient hospitalisation costs included but no description provided. | No, state only direct medical costs included and clearly state breakdown of hospitalisation costs. | No. | No, authors use Linas 2011 hospitalisation costs. | No.  Hospitalisation costs breakdown do not state any overheads/operating costs. |
| **5.    Were costs and consequences measured accurately in appropriate physical units?** | | | | | |
| 5.1. Were any of the identified items omitted from measurement? If so, does this mean that they carried no weight in the subsequent analysis? | Yes  Excluded adverse reaction costs as very rare events.  Assumed no impact. | No | No | Yes.  Excluded MDR-TB due to low prevalence and secondary reactivation of TB. Also did not include patient data for those who did not attend second clinic visit for TST reading.  Assumed no impact. | No |
| 5.2. Were there any special circumstances (e.g., joint use of resources) that made measurement difficult? Were these circumstances handled appropriately? | No | No | No | No | No |
| **6.    Were the cost and consequences valued credibly?** | | | | | |
| 6.1. Were the sources of all values clearly identified? | Yes | Yes | Yes | Yes but  no year of valuation stated. | Yes |
| 6.2. Were market values employed for changes involving resources gained or depleted? | Yes | Yes | Yes | Yes | Yes |
| 6.3. Where market values were absent (e.g. volunteer labour), or market values did not reflect actual values (such as clinic space donated at a reduced rate), were adjustments made to approximate market values? | N/A | N/A | N/A | N/A | N/A |
| 6.4. Was the valuation of consequences appropriate for the question posed? | Yes | Yes | Yes | Yes | Yes |
| **7.    Were costs and consequences adjusted for differential timing?** | | | | | |
| 7.1. Were costs and consequences that occur in the future ‘discounted’ to their present values? | Yes, 3%. | Yes, 3.5%. | No. | No. | Yes, 3%. |
| 7.2. Was there any justification given for the discount rate used? | Yes, state “standard rate” and referenced. | Yes, reference NICE recommendations. | N/A | N/A | No. |
| **8.    Was an incremental analysis of costs and consequences of alternatives performed?** | | | | | |
| 8.1. Were the additional (incremental) costs generated by one alternative over another compared to the additional effects, benefits, or utilities generated? | Yes | Yes | Yes | Yes | Yes |
| **9.    Was allowance made for uncertainty in the estimates of costs and consequences?** | | | | | |
| 9.1. If data on costs and consequences were stochastic (randomly determined sequence of observations), were appropriate statistical analyses performed? | Yes | Yes | Yes | Yes | Yes |
| 9.2. If sensitivity analysis was employed, was justification provided for the range of values (or for key study parameters)? | No  Stated “reasonable range” but no reference. | Yes | Yes | Yes | Yes |
| 9.3. Were the study results sensitive to changes in the values? | Yes  Sensitive to LTBI rate. | Yes  Sensitive to specificity of QFT-GIT. | Yes  Sensitive to QFT costs and TST specificity. | No | Yes  Sensitive to  TB re-activation rate. |
| **10.    Did the presentation and discussion of study results include all issues of concern to users?** | | | | | |
| 10.1. Were the conclusions of the analysis based on some overall index or ratio of costs to consequences (e.g. cost-effectiveness ratio)? | Yes | Yes | Yes | No  No cost-effectiveness ratio was used. Conclusions were based on “highest benefits” and “lowest cost” strategy. | Yes |
| 10.2. Were the results compared with those of others who have investigated the same question? If so, were allowances made for potential differences in study methodology? | No | Yes | Yes | No | No |
| 10.3. Did the study discuss the generalisability of the results to other settings and patient/client groups? | No | Yes | Yes | Yes | Yes |
| 10.4. Did the study allude to, or take account of, other important factors in the choice or decision under consideration (e.g. distribution of costs and consequences, or relevant ethical issues)? | Yes | Yes | Yes | Yes | Yes |
| 10.5. Did the study discuss issues of implementation, such as the feasibility of adopting the ‘preferred’ programme given existing financial or other constraints, and whether any freed resources could be redeployed to other worthwhile programmes? | Yes | Yes | Yes | No | No |

Continuation of **Table G**

| **Drummond Checklist Questions** | **Capocci, (2015)** | **Wingate (2015)** | **Auguste. (2016)** | **Nijhawan (2016)** | **Campbell (2017)** |
| --- | --- | --- | --- | --- | --- |
| 1. **Was a well-defined question posed in answerable form?** | | | | | |
| 1.1. Did the study examine both costs and effects of the service(s) or programme(s)? | Yes | Yes | Yes | Yes | Yes |
| 1.2. Did the study involve a comparison of alternatives? | Yes | Yes | Yes | Yes | Yes |
| 1.3. Was a viewpoint for the analysis stated and was the study placed in any decision-making context? | Yes  Adults living with HIV in the UK. | Yes  Pre arrival refugees to USA. | Yes  Children, immunocompromised people and recently Arrived immigrants and General Population in the UK. | Yes  Adults entering jail in the USA. | Yes  Pre-arrival refugees to Canada. |
| **2. Was a comprehensive description of the competing alternatives given?** | | | | | |
| 2.1. Were there any important alternatives omitted? | No | No | No | No | No |
| 2.2. Was (should) a do-nothing alternative be considered? | Yes, do nothing was included appropriately. | Yes, do nothing was included appropriately. | No | No | Yes, do nothing was included appropriately. |
| **3.    Was the effectiveness of the programme or services established?** | | | | | |
| 3.1. Was this done through a randomised, controlled clinical trial? If so, did the trial protocol reflect what would happen in regular practice? | No | No | No | No | No |
| 3.2. Was effectiveness established through an overview of clinical studies? | Somewhat.  Sensitivity of IGRA used from one clinical study. | Yes  Sensitivity and specificity of TST values from number of clinical studies | Yes  Systematic review carried out to establish sensitivity and specificity of tests. | Yes  Sensitivity and specificity of test values from clinical studies and systematic review. | Yes  Sensitivity and specificity of test values from clinical studies and systematic reviews. |
| 3.3. Were observational data or assumptions used to establish effectiveness? If so, what are the potential biases in results? | Yes  Both observational data from clinic and referenced assumptions used.  Unclear method of calculating effectiveness outcomes. | Yes  Key assumption is proportions of population with/without BCG vaccination with adequate references/reasoning. Authors use this assumption and one clinical study to estimate TST sensitivity. | Yes  Many assumptions for effectiveness with no references/reasoning. | No | Yes  Many assumptions for effectiveness all with adequate references/reasoning. |
| **4. Were all the important and relevant costs and consequences for each alternative identified?** | | | | | |
| 4.1. Was the range wide enough for the research question at hand? | Yes | Yes | Yes | Yes | Yes |
| 4.2. Did it cover all relevant viewpoints? | Yes | Yes | Yes | Yes | Yes |
| 4.3. Were the capital costs, as well as operating costs, included? | Unclear breakdown of costs provided. | No.  Includes labour but no indication of other operating/capital costs. | No.  Clear breakdown provided and capital/overheads not included. | Yes.  For QFT-GIT laboratory operations included. | No. |
| **5.    Were costs and consequences measured accurately in appropriate physical units?** | | | | | |
| 5.1. Were any of the identified items omitted from measurement? If so, does this mean that they carried no weight in the subsequent analysis? | No | No | No | Yes  Treatment and outcome costs.  Potential underestimate of benefits of QFT-GIT. | No |
| 5.2. Were there any special circumstances (e.g., joint use of resources) that made measurement difficult? Were these circumstances handled appropriately? | No | No | No | No | No |
| **6.    Were the cost and consequences valued credibly?** | | | | | |
| 6.1. Were the sources of all values clearly identified? | Yes | Yes | Yes | Yes | Yes |
| 6.2. Were market values employed for changes involving resources gained or depleted? | Yes | Yes | Yes | Yes | Yes |
| 6.3. Where market values were absent (e.g. volunteer labour), or market values did not reflect actual values (such as clinic space donated at a reduced rate), were adjustments made to approximate market values? | N/A | N/A | N/A | N/A | N/A |
| 6.4. Was the valuation of consequences appropriate for the question posed? | Yes | Yes | Yes | Yes | Yes |
| **7.    Were costs and consequences adjusted for differential timing?** | | | | | |
| 7.1. Were costs and consequences that occur in the future ‘discounted’ to their present values? | Yes, 3.5% | Yes, 3% | Yes, 3.5% | No | Yes, 1.5% |
| 7.2. Was there any justification given for the discount rate used? | Yes, reference NICE recommendations. | Yes, reference Haddix et al. 2003. | Yes, reference NICE recommendations. | N/A | Yes, reference Canadian Agency for Drugs and Technologies in Health recommendations. |
| **8.    Was an incremental analysis of costs and consequences of alternatives performed?** | | | | | |
| 8.1. Were the additional (incremental) costs generated by one alternative over another compared to the additional effects, benefits, or utilities generated? | Yes | Yes | Yes | Yes | Yes |
| **9.    Was allowance made for uncertainty in the estimates of costs and consequences?** | | | | | |
| 9.1. If data on costs and consequences were stochastic (randomly determined sequence of observations), were appropriate statistical analyses performed? | Yes | Yes | Yes | Yes | Yes |
| 9.2. If sensitivity analysis was employed, was justification provided for the range of values (or for key study parameters)? | Yes | Yes | Yes | No | Yes |
| 9.3. Were the study results sensitive to changes in the values? | No | No | No | No | Yes  Sensitive to treatments received. |
| **10.    Did the presentation and discussion of study results include all issues of concern to users?** | | | | | |
| 10.1. Were the conclusions of the analysis based on some overall index or ratio of costs to consequences (e.g. cost-effectiveness ratio)? | Yes | Yes | Yes | No | Yes |
| 10.2. Were the results compared with those of others who have investigated the same question? If so, were allowances made for potential differences in study methodology? | No | Yes | Yes | Yes | Yes |
| 10.3. Did the study discuss the generalisability of the results to other settings and patient/client groups? | Yes | No | Yes | Yes | Yes |
| 10.4. Did the study allude to, or take account of, other important factors in the choice or decision under consideration (e.g. distribution of costs and consequences, or relevant ethical issues)? | Yes | No | Yes | Yes | Yes |
| 10.5. Did the study discuss issues of implementation, such as the feasibility of adopting the ‘preferred’ programme given existing financial or other constraints, and whether any freed resources could be redeployed to other worthwhile programmes? | Yes | No | Yes | Yes | Yes |

Continuation of **Table G**

| **Drummond Checklist Questions** | **Haukaas (2017)** | **Mullie (2017)** | **Tasillo, (2017)** | **Abubakar (2018)** | **Li (2018)** |
| --- | --- | --- | --- | --- | --- |
| **1. Was a well-defined question posed in answerable form?** | | | | | |
| 1.1. Did the study examine both costs and effects of the service(s) or programme(s)? | Yes | Yes | Yes | Yes | Yes |
| 1.2. Did the study involve a comparison of alternatives? | Yes | Yes | Yes | Yes | Yes |
| 1.3. Was a viewpoint for the analysis stated and was the study placed in any decision-making context? | Yes  Recently arrived immigrants <35y.o to Norway | Yes  HCW with negative TST at time of employment. | Yes  US-born or migrants living with or without comorbidities. | Yes  Recent immigrants and contacts in the UK. | Yes  Elderly (>65y.o.) at admission to residential care home in Hong Kong. |
| **2. Was a comprehensive description of the competing alternatives given?** | | | | | |
| 2.1. Were there any important alternatives omitted? | No | No | No | No | No |
| 2.2. Was (should) a do-nothing alternative be considered? | Yes, do nothing was included appropriately. | No | Yes, do nothing was included appropriately. | Yes, do nothing was included appropriately. | Yes, do nothing was included appropriately. |
| **3.    Was the effectiveness of the programme or services established?** | | | | | |
| 3.1. Was this done through a randomised, controlled clinical trial? If so, did the trial protocol reflect what would happen in regular practice? | No | No | No |  | No |
| 3.2. Was effectiveness established through an overview of clinical studies? | Somewhat.  Sensitivity and specificity of TST values from only one study. | Yes  Sensitivity and specificity of tests values from number of clinical studies. | Yes  Sensitivity and specificity of tests values from number of clinical studies. | No | Yes  Sensitivity and specificity of tests values from number of clinical studies. |
| 3.3. Were observational data or assumptions used to establish effectiveness? If so, what are the potential biases in results? | Yes  Expert opinion and estimation used for multiple key effectiveness parameter.  Other assumptions provided with no references/reasoning. | Yes  Key assumption is no loss of QALYs with uncomplicated treatment of LTBI.  All other assumptions references/reasoned adequately. | Yes  Key assumption is that LTBI treatment without adverse effects causes no change in quality of life.  Other assumptions provided with no references/reasoning. | Yes  Cohort trial used to estimate sensitivity and specificity of tests.  Reporting and selection bias possible. | Yes  Many assumptions for effectiveness all with adequate references/reasoning. |
| **4. Were all the important and relevant costs and consequences for each alternative identified?** | | | | | |
| 4.1. Was the range wide enough for the research question at hand? | Yes | Yes | Yes | Yes | Yes |
| 4.2. Did it cover all relevant viewpoints? | Yes | Yes | Yes | Yes | Yes |
| 4.3. Were the capital costs, as well as operating costs, included? | Unclear.  Hospitalisation costs included but no breakdown provided. | Unclear.  Hospitalisation costs included but no breakdown provided. | Unclear.  Treatment costs included but no breakdown provided. | No.  Clear breakdown provided and capital/overheads not included | No.  Clear breakdown provided and capital/overheads not included |
| **5.    Were costs and consequences measured accurately in appropriate physical units?** | | | | | |
| 5.1. Were any of the identified items omitted from measurement? If so, does this mean that they carried no weight in the subsequent analysis? | Yes  VAT, MDR-TB assumed minimal effect on analysis. | No | No | No | Yes.  Costs of minor adverse events or additional radiologic tests.  Underestimation of costs. |
| 5.2. Were there any special circumstances (e.g., joint use of resources) that made measurement difficult? Were these circumstances handled appropriately? | No | No | No | No | No |
| **6.    Were the cost and consequences valued credibly?** | | | | | |
| 6.1. Were the sources of all values clearly identified? | Yes | Yes | Yes | Yes, but year of valuation not stated. | Yes, but year of valuation not stated. |
| 6.2. Were market values employed for changes involving resources gained or depleted? | Yes | Yes | Yes | Yes | Yes |
| 6.3. Where market values were absent (e.g. volunteer labour), or market values did not reflect actual values (such as clinic space donated at a reduced rate), were adjustments made to approximate market values? | N/A | N/A | N/A | N/A | N/A |
| 6.4. Was the valuation of consequences appropriate for the question posed? | Yes | Yes | Yes | Yes | Yes |
| **7.    Were costs and consequences adjusted for differential timing?** | | | | | |
| 7.1. Were costs and consequences that occur in the future ‘discounted’ to their present values? | Yes, 4%  Only costs discounted in base case analysis. | Yes, 3% | Yes, 3% | Yes, 3.5% | Yes, 5% |
| 7.2. Was there any justification given for the discount rate used? | Yes, reference Norwegian Directorate of Health recommendations. | Yes, reference Sanders et al. 2016 recommendations. | State current recommendations. | State current recommendations. | Yes, reference Drummond et al. 2005. |
| **8.    Was an incremental analysis of costs and consequences of alternatives performed?** | | | | | |
| 8.1. Were the additional (incremental) costs generated by one alternative over another compared to the additional effects, benefits, or utilities generated? | Yes | Yes | Yes | Yes | Yes |
| **9.    Was allowance made for uncertainty in the estimates of costs and consequences?** | | | | | |
| 9.1. If data on costs and consequences were stochastic (randomly determined sequence of observations), were appropriate statistical analyses performed? | Yes | Yes | Yes | Yes | Yes |
| 9.2. If sensitivity analysis was employed, was justification provided for the range of values (or for key study parameters)? | No | Yes | Yes | Yes | Yes |
| 9.3. Were the study results sensitive to changes in the values? | Yes.  Sensitive to IGRA cost. | No | Yes  Sensitive to LTBI rate and TST sensitivity. | No | No. |
| **10.    Did the presentation and discussion of study results include all issues of concern to users?** | | | | | |
| 10.1. Were the conclusions of the analysis based on some overall index or ratio of costs to consequences (e.g. cost-effectiveness ratio)? | Yes | Yes | Yes | Yes | Yes |
| 10.2. Were the results compared with those of others who have investigated the same question? If so, were allowances made for potential differences in study methodology? | Yes | Yes | No | No | Yes |
| 10.3. Did the study discuss the generalisability of the results to other settings and patient/client groups? | No | Yes | No | Yes | Yes |
| 10.4. Did the study allude to, or take account of, other important factors in the choice or decision under consideration (e.g. distribution of costs and consequences, or relevant ethical issues)? | Yes | Yes | Yes | Yes | Yes |
| 10.5. Did the study discuss issues of implementation, such as the feasibility of adopting the ‘preferred’ programme given existing financial or other constraints, and whether any freed resources could be redeployed to other worthwhile programmes? | Yes | No | No | Yes | Yes |

Continuation of **Table G**

| **Drummond Checklist Questions** | **Sohn (2018).** | **Campbell (2019a).** | **Campbell (2019b).** | **Loureiro (2019).** | **Png (2019).** |
| --- | --- | --- | --- | --- | --- |
| 1. **Was a well-defined question posed in answerable form?** | | | | | |
| 1.1. Did the study examine both costs and effects of the service(s) or programme(s)? | Yes | Yes | Yes | Yes | Yes |
| 1.2. Did the study involve a comparison of alternatives? | Yes | Yes | Yes | Yes | Yes |
| 1.3. Was a viewpoint for the analysis stated and was the study placed in any decision-making context? | Yes  Adolescents (13-18y.o.) contacts in Japan. | Yes  Pre-arrival immigrants to Canada | Yes  Migrants with either late-stage CKD or beginning dialysis. | Yes  Primary HCW in Brazil. | Yes  HCW in Singapore. |
| **2. Was a comprehensive description of the competing alternatives given?** | | | | | |
| 2.1. Were there any important alternatives omitted? | No | No | No | No | No |
| 2.2. Was (should) a do-nothing alternative be considered? | No | Yes | Yes, do nothing was included appropriately. | No | Yes, do nothing was included appropriately. |
| **3.    Was the effectiveness of the programme or services established?** | | | | | |
| 3.1. Was this done through a randomised, controlled clinical trial? If so, did the trial protocol reflect what would happen in regular practice? | No | No | No | No | No |
| 3.2. Was effectiveness established through an overview of clinical studies? | Yes  Sensitivity and specificity of tests values from number of clinical studies and systematic reviews. | Yes  Sensitivity and specificity of tests values from number of clinical studies. | Yes  Sensitivity and specificity of tests values from number of clinical studies. | Yes  Sensitivity and specificity of tests values from number of clinical studies. | Yes |
| 3.3. Were observational data or assumptions used to establish effectiveness?  If so, what are the potential biases in results? | Yes  Key assumption is the proportion of recent infection among contacts with LTBI.  All assumptions for effectiveness all have adequate references/reasoning. | Yes  All assumptions for effectiveness all have adequate references/reasoning. | Yes  Key assumption is that no individuals had active TB at the time of LTBI screening.  All assumptions for effectiveness all have adequate references/reasoning. | Yes  Multiple assumptions for effectiveness all with no references/reasoning. | Yes  Assumed 100% specificity and sensitivity of QFT-G with reasoning and references.  All assumptions are stated to be from “published literature or expert opinion” |
| **4. Were all the important and relevant costs and consequences for each alternative identified?** | | | | | |
| 4.1. Was the range wide enough for the research question at hand? | Yes | Yes | Yes | Yes | Yes |
| 4.2. Did it cover all relevant viewpoints? | Yes | Yes | Yes | Yes | Yes |
| 4.3. Were the capital costs, as well as operating costs, included? | Yes.  Includes overheads and vehicle operations. | Unclear.  Hospitalisation costs included but no breakdown provided. | Unclear.  Hospitalisation costs included but no breakdown provided. | Yes.  No capital costs stated but equipment use costs clearly included. | Yes.  State that hospitalisation and testing costs include labour and overhead costs. |
| **5.    Were costs and consequences measured accurately in appropriate physical units?** | | | | | |
| 5.1. Were any of the identified items omitted from measurement?  If so, does this mean that they carried no weight in the subsequent analysis? | Yes  Cost of referral to clinic assumed relatively minimal impact. | No | No | Yes  MDR-TB due to low prevalence.  Assumed minimal impact. | No |
| 5.2. Were there any special circumstances (e.g., joint use of resources) that made measurement difficult? Were these circumstances handled appropriately? | No | No | No | No | No |
| **6.    Were the cost and consequences valued credibly?** | | | | | |
| 6.1. Were the sources of all values clearly identified? | Yes | Yes | Yes | No | Yes |
| **6.2. Were market values employed for changes involving resources gained or depleted?** | Yes | Yes | Yes | No | Yes |
| 6.3. Where market values were absent (e.g. volunteer labour), or market values did not reflect actual values (such as clinic space donated at a reduced rate), were adjustments made to approximate market values? | N/A | N/A | N/A | N/A | N/A |
| 6.4. Was the valuation of consequences appropriate for the question posed? | Yes | Yes | Yes | Yes | Yes |
| **7.    Were costs and consequences adjusted for differential timing?** | | | | | |
| 7.1. Were costs and consequences that occur in the future ‘discounted’ to their present values? | No | Yes, 3% | Yes, 1.5% | No | Yes, 3% |
| 7.2. Was there any justification given for the discount rate used? | State due to short horizon of analysis. | Yes, reference Sanders et al. 2016. | Yes, reference Canadian Agency for Drugs and Technologies in Health recommendations. | State due to short horizon of analysis. | Only state “commonly used rate”. |
| **8.    Was an incremental analysis of costs and consequences of alternatives performed?** | | | | | |
| 8.1. Were the additional (incremental) costs generated by one alternative over another compared to the additional effects, benefits, or utilities generated? | Yes | Yes | Yes | Yes | Yes |
| **9.    Was allowance made for uncertainty in the estimates of costs and consequences?** | | | | | |
| 9.1. If data on costs and consequences were stochastic (randomly determined sequence of observations), were appropriate statistical analyses performed? | Yes | Yes | Yes | Yes | Yes |
| 9.2. If sensitivity analysis was employed, was justification provided for the range of values (or for key study parameters)? | Yes | Yes | Yes | Yes | Yes |
| 9.3. Were the study results sensitive to changes in the values? | Yes  Sensitive to QFT-GIT cost. | Yes  For migrants from low incidence conclusion only cost-effective 50% of time in probabilistic sensitivity analysis. | No | No | No |
| **10.    Did the presentation and discussion of study results include all issues of concern to users?** | | | | | |
| 10.1. Were the conclusions of the analysis based on some overall index or ratio of costs to consequences (e.g. cost-effectiveness ratio)? | Yes | Yes | Yes | Yes | Yes |
| 10.2. Were the results compared with those of others who have investigated the same question? If so, were allowances made for potential differences in study methodology? | Yes | Yes | Yes | Yes | Yes |
| 10.3. Did the study discuss the generalisability of the results to other settings and patient/client groups? | Yes | Yes | Yes | No | Yes |
| 10.4. Did the study allude to, or take account of, other important factors in the choice or decision under consideration (e.g. distribution of costs and consequences, or relevant ethical issues)? | Yes | Yes | Yes | Yes | No |
| 10.5. Did the study discuss issues of implementation, such as the feasibility of adopting the ‘preferred’ programme given existing financial or other constraints, and whether any freed resources could be redeployed to other worthwhile programmes? | Yes | Yes | Yes | Yes | Yes |

Continuation of **Table G**

| **Drummond Checklist Questions** | | **Al Abri (2020).** | **Jo (2020).** | **Steffen (2020)** | **Kim (2018)** |
| --- | --- | --- | --- | --- | --- |
| **1.Was a well-defined question posed in answerable form?** | | | | |  |
| 1.1. Did the study examine both costs and effects of the service(s) or programme(s)? | Yes | | Yes | Yes | Yes |
| 1.2. Did the study involve a comparison of alternatives? | Yes | | Yes | Yes | Yes |
| 1.3. Was a viewpoint for the analysis stated and was the study placed in any decision-making context? | Yes  20-year-old recent immigrants in Oman. | | Yes  General population with vulnerable sub-populations. | Yes  Adults living with HIV in Brazil. | Yes  HIV+ Pregnant women |
| **2. Was a comprehensive description of the competing alternatives given?** | | | | |  |
| 2.1. Were there any important alternatives omitted? | No | | Unclear alternatives provided. | No | No |
| 2.2. Was (should) a do-nothing alternative be considered? | No | | No | No | No |
| **3.    Was the effectiveness of the programme or services established?** | | | | |  |
| 3.1. Was this done through a randomised, controlled clinical trial? If so, did the trial protocol reflect what would happen in regular practice? | No | | No | No | No |
| 3.2. Was effectiveness established through an overview of clinical studies? | Yes  Sensitivity and specificity of tests values from number of clinical studies and systematic reviews. | | Yes  Sensitivity and tests values from a single systematic review. | Yes  Sensitivity and specificity of tests values from number of systematic reviews. | Yes  Sensitivity and specificity of tests values from number of systematic reviews. |
| 3.3. Were observational data or assumptions used to establish effectiveness?  If so, what are the potential biases in results? | Yes  No clear assumptions stated.  TB incidence and the LTBI rate, BCG vaccination rate, (MDR-TB) rate, TB mortality, and rate of adherence to treatment were imputed from Omani data. | | Yes  Most assumptions for effectiveness all have adequate references/reasoning. | Yes  All assumptions for effectiveness all have adequate references/reasoning. | Yes  All assumptions for effectiveness all have adequate references/reasoning. |
| **4. Were all the important and relevant costs and consequences for each alternative identified?** | | | | |  |
| 4.1. Was the range wide enough for the research question at hand? | | Yes | No | Yes | Yes |
| 4.2. Did it cover all relevant viewpoints? | | Yes | Yes | Yes | Yes |
| 4.3. Were the capital costs, as well as operating costs, included? | | Unclear breakdown of costs provided. | Unclear breakdown of costs provided. | Yes.  No capital costs stated but equipment use costs clearly included. | Yes  Costs of overheads, building space, equipment, staff, and consumables included. |
| **5.    Were costs and consequences measured accurately in appropriate physical units?** | | | | |  |
| 5.1. Were any of the identified items omitted from measurement?  If so, does this mean that they carried no weight in the subsequent analysis? | | No | No | No | No |
| 5.2. Were there any special circumstances (e.g., joint use of resources) that made measurement difficult? Were these circumstances handled appropriately? | | No | No | No | No |
| **6.    Were the cost and consequences valued credibly?** | | | | |  |
| 6.1. Were the sources of all values clearly identified? | | No | Yes | Yes | Yes |
| **6.2. Were market values employed for changes involving resources gained or depleted?** | | No | Yes | Yes | Yes |
| 6.3. Where market values were absent (e.g. volunteer labour), or market values did not reflect actual values (such as clinic space donated at a reduced rate), were adjustments made to approximate market values? | | N/A | N/A | N/A | N/A |
| 6.4. Was the valuation of consequences appropriate for the question posed? | | No | Yes | Yes | Yes |
| **7.    Were costs and consequences adjusted for differential timing?** | | | | |  |
| 7.1. Were costs and consequences that occur in the future ‘discounted’ to their present values? | | No | Yes, 3% | Yes, 3% | Yes, 3% |
| 7.2. Was there any justification given for the discount rate used? | | No | Yes, reference Sassi 2006. | Yes, reference Brazilian Ministry of Health recommendations. | No |
| **8.    Was an incremental analysis of costs and consequences of alternatives performed?** | | | | |  |
| 8.1. Were the additional (incremental) costs generated by one alternative over another compared to the additional effects, benefits, or utilities generated? | | Yes | Yes | Yes | Yes |
| **9.    Was allowance made for uncertainty in the estimates of costs and consequences?** | | | | |  |
| 9.1. If data on costs and consequences were stochastic (randomly determined sequence of observations), were appropriate statistical analyses performed? | | Yes | Yes | Yes | Yes |
| 9.2. If sensitivity analysis was employed, was justification provided for the range of values (or for key study parameters)? | | No | Yes | Yes | Yes |
| 9.3. Were the study results sensitive to changes in the values? | | Yes  Sensitive to treatments received. | Yes  Specificity of QFT-GIT. | Yes  Cost of DiaskinTest. | Yes  Sensitive to the probability of developing TB and LTBI prevalence |
| **10.    Did the presentation and discussion of study results include all issues of concern to users?** | | | | |  |
| 10.1. Were the conclusions of the analysis based on some overall index or ratio of costs to consequences (e.g. cost-effectiveness ratio)? | | Yes | Yes | Yes | Yes |
| 10.2. Were the results compared with those of others who have investigated the same question? If so, were allowances made for potential differences in study methodology? | | Yes | Yes | Yes | Yes |
| 10.3. Did the study discuss the generalisability of the results to other settings and patient/client groups? | | No | Yes | Yes | Yes |
| 10.4. Did the study allude to, or take account of, other important factors in the choice or decision under consideration (e.g. distribution of costs and consequences, or relevant ethical issues)? | | No | Yes | Yes | Yes |
| 10.5. Did the study discuss issues of implementation, such as the feasibility of adopting the ‘preferred’ programme given existing financial or other constraints, and whether any freed resources could be redeployed to other worthwhile programmes? | | No | Yes | Yes | Yes |

Continuation of **Table G**

| **Drummond Checklist Questions** | **Auguste (2022)** | **Fekadu (2022)** | **Marx (2021)** |
| --- | --- | --- | --- |
| **1.Was a well-defined question posed in answerable form?** | | | |
| 1.1. Did the study examine both costs and effects of the service(s) or programme(s)? | Yes | Yes | Yes |
| 1.2. Did the study involve a comparison of alternatives? | Yes | Yes | Yes |
| 1.3. Was a viewpoint for the analysis stated and was the study placed in any decision-making context? | Yes  Adults living with HIV | Yes  Adults living with HIV | Yes  Asylum Seekers in Germany |
| **2. Was a comprehensive description of the competing alternatives given?** | | | |
| 2.1. Were there any important alternatives omitted? | No | No | No |
| 2.2. Was (should) a do-nothing alternative be considered? | Yes | Yes | Yes |
| **3.    Was the effectiveness of the programme or services established?** | | | |
| 3.1. Was this done through a randomised, controlled clinical trial? If so, did the trial protocol reflect what would happen in regular practice? | No | No | No |
| 3.2. Was effectiveness established through an overview of clinical studies? | Yes  Sensitivity and specificity of tests values from number of clinical studies and systematic reviews. | Yes  Sensitivity and tests values from a single systematic review. | Yes  Sensitivity and specificity of tests values from number of systematic reviews. |
| 3.3. Were observational data or assumptions used to establish effectiveness?  If so, what are the potential biases in results? | Yes  Most assumptions for effectiveness all have adequate references/reasoning. | Yes  Most assumptions for effectiveness all have adequate references/reasoning. | Yes  Most assumptions for effectiveness all have adequate references/reasoning. |
| **4. Were all the important and relevant costs and consequences for each alternative identified?** | | | |
| 4.1. Was the range wide enough for the research question at hand? | Yes | Yes | Yes |
| 4.2. Did it cover all relevant viewpoints? | Yes | Yes | Yes |
| 4.3. Were the capital costs, as well as operating costs, included? | No breakdown of costs. | No breakdown of costs. | No breakdown of costs. |
| **5.    Were costs and consequences measured accurately in appropriate physical units?** | | | |
| 5.1. Were any of the identified items omitted from measurement?  If so, does this mean that they carried no weight in the subsequent analysis? | No | No | No |
| 5.2. Were there any special circumstances (e.g., joint use of resources) that made measurement difficult? Were these circumstances handled appropriately? | No | No | No |
| **6.    Were the cost and consequences valued credibly?** | | | |
| 6.1. Were the sources of all values clearly identified? | Yes | Yes | Yes |
| **6.2. Were market values employed for changes involving resources gained or depleted?** | Yes | Yes | Yes |
| 6.3. Where market values were absent (e.g. volunteer labour), or market values did not reflect actual values (such as clinic space donated at a reduced rate), were adjustments made to approximate market values? | N/A | N/A | N/A |
| 6.4. Was the valuation of consequences appropriate for the question posed? | Yes | Yes | Yes |
| **7.    Were costs and consequences adjusted for differential timing?** | | | |
| 7.1. Were costs and consequences that occur in the future ‘discounted’ to their present values? | No | Yes | Yes |
| 7.2. Was there any justification given for the discount rate used? | No | No | No |
| **8.    Was an incremental analysis of costs and consequences of alternatives performed?** | | | |
| 8.1. Were the additional (incremental) costs generated by one alternative over another compared to the additional effects, benefits, or utilities generated? | Yes | Yes | Yes |
| **9.    Was allowance made for uncertainty in the estimates of costs and consequences?** | | | |
| 9.1. If data on costs and consequences were stochastic (randomly determined sequence of observations), were appropriate statistical analyses performed? | Yes | Yes | Yes |
| 9.2. If sensitivity analysis was employed, was justification provided for the range of values (or for key study parameters)? | No | No | No |
| 9.3. Were the study results sensitive to changes in the values? | Yes | Yes | Yes |
| **10.    Did the presentation and discussion of study results include all issues of concern to users?** | | | |
| 10.1. Were the conclusions of the analysis based on some overall index or ratio of costs to consequences (e.g. cost-effectiveness ratio)? | Yes | Yes | Yes |
| 10.2. Were the results compared with those of others who have investigated the same question? If so, were allowances made for potential differences in study methodology? | Yes | Yes | Yes |
| 10.3. Did the study discuss the generalisability of the results to other settings and patient/client groups? | Yes | Yes | Yes |
| 10.4. Did the study allude to, or take account of, other important factors in the choice or decision under consideration (e.g. distribution of costs and consequences, or relevant ethical issues)? | Yes | Yes | Yes |
| 10.5. Did the study discuss issues of implementation, such as the feasibility of adopting the ‘preferred’ programme given existing financial or other constraints, and whether any freed resources could be redeployed to other worthwhile programmes? | Yes | Yes | Yes |

# **VI. Summary of Drummond’s checklist**

**Table H.** Summary of the proportion of articles accomplishing each of the Drummond’s criteria

| **Drummond Checklist Questions** | **% Of papers accomplishing each criterion** | | |
| --- | --- | --- | --- |
|  | **All articles** | **DiaskinTest-related** | **TST or IGRA** |
| **1.     Was a well-defined question posed in answerable form?** |  |  |  |
| 1.1. Did the study examine both costs and effects of the service(s) or programme(s)? | 97.1% | 87.5% | 100.0% |
| 1.2. Did the study involve a comparison of alternatives? | 100.0% | 100.0% | 100.0% |
| 1.3. Was a viewpoint for the analysis stated and was the study placed in any decision-making context? | 100.0% | 100.0% | 100.0% |
| **2. Was a comprehensive description of the competing alternatives given?** |  |  |  |
| 2.1. Were there any important alternatives omitted? | 5.7% | 25.0% | 0.0% |
| 2.2. Was (should) a do-nothing alternative be considered? | 42.9% | 12.5% | 50.0% |
| **3.    Was the effectiveness of the programme or services established?** |  |  |  |
| 3.1. Was this done through a randomised, controlled clinical trial? If so, did the trial protocol reflect what would happen in regular practice? | 0.0% | 0.0% | 0.0% |
| 3.2. Was effectiveness established through an overview of clinical studies? | 91.4% | 87.5% | 92.9% |
| 3.3. Were observational data or assumptions used to establish effectiveness? If so, what are the potential biases in results? | 85.7% | 62.5% | 92.9% |
| **4. Were all the important and relevant costs and consequences for each alternative identified?** |  |  |  |
| 4.1. Was the range wide enough for the research question at hand? | 97.1% | 100.0% | 96.4% |
| 4.2. Did it cover all relevant viewpoints? | 100.0% | 100.0% | 100.0% |
| 4.3. Were the capital costs, as well as operating costs, included? | 31.4% | 62.5% | 25.0% |
| **5.    Were costs and consequences measured accurately in appropriate physical units?** |  |  |  |
| 5.1. Were any of the identified items omitted from measurement? If so, does this mean that they carried no weight in the subsequent analysis? | 25.7% | 25.0% | 25.0% |
| 5.2. Were there any special circumstances (e.g., joint use of resources) that made measurement difficult? Were these circumstances handled appropriately? | 0.0% | 0.0% | 0.0% |
| **6.    Were the cost and consequences valued credibly?** |  |  |  |
| 6.1. Were the sources of all values clearly identified? | 82.9% | 50.0% | 92.9% |
| 6.2. Were market values employed for changes involving resources gained or depleted? | 94.3% | 100.0% | 92.9% |
| 6.3. Where market values were absent (e.g. volunteer labour), or market values did not reflect actual values (such as clinic space donated at a reduced rate), were adjustments made to approximate market values? | 0.0% | 0.0% | 0.0% |
| 6.4. Was the valuation of consequences appropriate for the question posed? | 94.3% | 87.5% | 96.4% |
| **7.    Were costs and consequences adjusted for differential timing?** |  |  |  |
| 7.1. Were costs and consequences that occur in the future ‘discounted’ to their present values? | 60.0% | 12.5% | 75.0% |
| 7.2. Was there any justification given for the discount rate used? | 60.0% | 12.5% | 75.0% |
| **8.    Was an incremental analysis of costs and consequences of alternatives performed?** |  |  |  |
| 8.1. Were the additional (incremental) costs generated by one alternative over another compared to the additional effects, benefits, or utilities generated? | 94.3% | 75.0% | 100.0% |
| **9.    Was allowance made for uncertainty in the estimates of costs and consequences?** |  |  |  |
| 9.1. If data on costs and consequences were stochastic (randomly determined sequence of observations), were appropriate statistical analyses performed? | 77.1% | 37.5% | 89.3% |
| 9.2. If sensitivity analysis was employed, was justification provided for the range of values (or for key study parameters)? | 71.4% | 37.5% | 82.1% |
| 9.3. Were the study results sensitive to changes in the values? | 34.3% | 12.5% | 42.9% |
| **10. Did the presentation and discussion of study results include all issues of concern to users?** |  |  |  |
| 10.1. Were the conclusions of the analysis based on some overall index or ratio of costs to consequences (e.g. cost-effectiveness ratio)? | 77.1% | 50.0% | 85.7% |
| 10.2. Were the results compared with those of others who have investigated the same question? If so, were allowances made for potential differences in study methodology? | 40.0% | 12.5% | 50.0% |
| 10.3. Did the study discuss the generalisability of the results to other settings and patient/client groups? | 57.1% | 37.5% | 64.3% |
| 10.4. Did the study allude to, or take account of, other important factors in the choice or decision under consideration (e.g. distribution of costs and consequences, or relevant ethical issues)? | 62.9% | 37.5% | 71.4% |
| 10.5. Did the study discuss issues of implementation, such as the feasibility of adopting the ‘preferred’ programme given existing financial or other constraints, and whether any freed resources could be redeployed to other worthwhile programmes? | 62.9% | 50.0% | 67.9% |

# **VII. References**

1. Куликов А, Зинчук И, Проценко М, Крысанов И. Диаскинтест для скрининга детей и подростков на туберкулезную инфекцию: подходы к ценообразованию и анализ затраты–эффективность. Туберкулез и болезни легких. 2009;9:41-6.

2. Аксенова ВА, Барышникова Л, Клевно НИ, Сокольская ЕА, Долженко Е, Мартьянов В, et al. Новые возможности скрининга и диагностики различных проявлений туберкулезной инфекции у детей и подростков в России. Вопросы современной педиатрии. 2011;10(4).

3. Ягудина Р, Зинчук И. Фармакоэкономическое исследование лекарственных средств для диагностики туберкулезной инфекции. Фармакоэкономика Современная фармакоэкономика и фармакоэпидемиология. 2013;6(1).

4. Солодун ИЮ, Эва ХМ, Башлакова ЕЕ, Ермолаева ТН, Давыдовская МВ, Евдошенко ЕП. Клинико-экономический анализ применения метода диагностики туберкулезной инфекции у детей и подростков с использованием аллергена туберкулезного рекомбинантного. Проблемы стандартизации в здравоохранении. 2017(3-4).

5. Чугаев Ю, Камаева Н, Цветков А, Кудлай Д, Черняев И. ИННОВАЦИОННЫЕ РЕКОМБИНАНТНЫЕ ТЕХНОЛОГИИ ВЫЯВЛЕНИЯ И ДИАГНОСТИКИ ТУБЕРКУЛЕЗА У ДЕТЕЙ И ПОДРОСТКОВ: ДОСТИЖЕНИЯ И ПРОБЛЕМЫ. Pediatriya named after GN Speransky. 2020;96(6).

6. Steffen RE, Pinto M, Kritski A, Trajman A. Cost-effectiveness of newer technologies for the diagnosis of Mycobacterium tuberculosis infection in Brazilian people living with HIV. Scientific reports. 2020;10(1):1-12.

7. Linas BP, Wong AY, Freedberg KA, Horsburgh Jr CR. Priorities for screening and treatment of latent tuberculosis infection in the United States. American journal of respiratory and critical care medicine. 2011;184(5):590-601.

8. Pareek M, Watson JP, Ormerod LP, Kon OM, Woltmann G, White PJ, et al. Screening of immigrants in the UK for imported latent tuberculosis: a multicentre cohort study and cost-effectiveness analysis. The Lancet infectious diseases. 2011;11(6):435-44.

9. del Campo MT, Fouad H, Solís-Bravo MM, Sánchez-Uriz MA, Mahíllo-Fernández I, Esteban J. Cost-effectiveness of different screening strategies (single or dual) for the diagnosis of tuberculosis infection in healthcare workers. Infection Control & Hospital Epidemiology. 2012;33(12):1226-34.

10. Eralp MN, Scholtes S, Martell G, Winter R, Exley AR. Screening of healthcare workers for tuberculosis: development and validation of a new health economic model to inform practice. BMJ open. 2012;2(2):e000630.

11. Shah M, Miele K, Choi H, DiPietro D, Martins-Evora M, Marsiglia V, et al. QuantiFERON-TB gold in-tube implementation for latent tuberculosis diagnosis in a public health clinic: a cost-effectiveness analysis. BMC infectious diseases. 2012;12(1):1-10.

12. Mandalakas AM, Hesseling AC, Gie RP, Schaaf H, Marais BJ, Sinanovic E. Modelling the cost-effectiveness of strategies to prevent tuberculosis in child contacts in a high-burden setting. Thorax. 2013;68(3):247-55.

13. Pareek M, Bond M, Shorey J, Seneviratne S, Guy M, White P, et al. Community-based evaluation of immigrant tuberculosis screening using interferon γ release assays and tuberculin skin testing: observational study and economic analysis. Thorax. 2013;68(3):230-9.

14. Steffen RE, Caetano R, Pinto M, Chaves D, Ferrari R, Bastos M, et al. Cost-effectiveness of Quantiferon®-TB Gold-in-Tube versus tuberculin skin testing for contact screening and treatment of latent tuberculosis infection in Brazil. PloS one. 2013;8(4):e59546.

15. Swaminath A, Bhadelia N, Wang YC. Cost-effectiveness of QuantiFERON testing before initiation of biological therapy in inflammatory bowel disease. Inflammatory bowel diseases. 2013;19(11):2444-9.

16. Verma G, Chuck A, Jacobs P. Tuberculosis screening for long-term care: a cost-effectiveness analysis. The International journal of tuberculosis and lung disease. 2013;17(9):1170-7.

17. Capocci S, Smith C, Morris S, Bhagani S, Cropley I, Abubakar I, et al. Decreasing cost effectiveness of testing for latent TB in HIV in a low TB incidence area. European Respiratory Journal. 2015;46(1):165-74.

18. La’Marcus TW, Coleman MS, de la Motte Hurst C, Semple M, Zhou W, Cetron MS, et al. A cost-benefit analysis of a proposed overseas refugee latent tuberculosis infection screening and treatment program. BMC public health. 2015;15(1):1-14.

19. Auguste P, Tsertsvadze A, Pink J, Seedat F, Gurung T, Freeman K, et al. Accurate diagnosis of latent tuberculosis in children, people who are immunocompromised or at risk from immunosuppression and recent arrivals from countries with a high incidence of tuberculosis: systematic review and economic evaluation. Health Technology Assessment. 2016;20(38):1-678.

20. Nijhawan AE, Iroh PA, Brown LS, Winetsky D, Porsa E. Cost analysis of tuberculin skin test and the QuantiFERON-TB Gold In-tube test for tuberculosis screening in a correctional setting in Dallas, Texas, USA. BMC infectious diseases. 2016;16(1):1-11.

21. Campbell JR, Johnston JC, Sadatsafavi M, Cook VJ, Elwood RK, Marra F. Cost-effectiveness of post-landing latent tuberculosis infection control strategies in new migrants to Canada. PloS one. 2017;12(10):e0186778.

22. Haukaas FS, Arnesen TM, Winje BA, Aas E. Immigrant screening for latent tuberculosis in Norway: a cost-effectiveness analysis. The European Journal of Health Economics. 2017:405-15.

23. Mullie GA, Schwartzman K, Zwerling A, N’Diaye DS. Revisiting annual screening for latent tuberculosis infection in healthcare workers: a cost-effectiveness analysis. BMC medicine. 2017;15(1):1-15.

24. Tasillo A, Salomon JA, Trikalinos TA, Horsburgh CR, Marks SM, Linas BP. Cost-effectiveness of testing and treatment for latent tuberculosis infection in residents born outside the United States with and without medical comorbidities in a simulation model. JAMA internal medicine. 2017;177(12):1755-64.

25. Abubakar I, Lalvani A, Southern J, Sitch A, Jackson C, Onyimadu O, et al. Two interferon gamma release assays for predicting active tuberculosis: the UK PREDICT TB prognostic test study. Health technology assessment (Winchester, England). 2018;22(56):1.

26. Li J, Yip BH, Leung C, Chung W, Kwok KO, Chan EY, et al. Screening for latent and active tuberculosis infection in the elderly at admission to residential care homes: a cost-effectiveness analysis in an intermediate disease burden area. PloS one. 2018;13(1):e0189531.

27. Sohn H, Kim H, Lee S. Cost-effectiveness of contact screening strategies for tuberculosis among high-school adolescents in South Korea. The International Journal of Tuberculosis and Lung Disease. 2018;22(5):496-503.

28. Campbell JR, Johnston JC, Cook VJ, Sadatsafavi M, Elwood RK, Marra F. Cost-effectiveness of latent tuberculosis infection screening before immigration to low-incidence countries. Emerging infectious diseases. 2019;25(4):661.

29. Campbell JR, Johnston JC, Ronald LA, Sadatsafavi M, Balshaw RF, Cook VJ, et al. Screening for latent tuberculosis infection in migrants with CKD: a cost-effectiveness analysis. American Journal of Kidney Diseases. 2019;73(1):39-50.

30. Loureiro RB, Maciel ELN, Caetano R, Peres RL, Fregona G, Golub JE, et al. Cost-effectiveness of QuantiFERON-TB Gold In-Tube versus tuberculin skin test for diagnosis and treatment of Latent Tuberculosis Infection in primary health care workers in Brazil. PloS one. 2019;14(11):e0225197.

31. Png ME, Yoong J, Ong CWM, Fisher D, Bagdasarian N. A screening strategy for latent tuberculosis in healthcare workers: Cost-effectiveness and budget impact of universal versus targeted screening. Infection Control & Hospital Epidemiology. 2019;40(3):341-9.

32. Al Abri S, Kowada A, Yaqoubi F, Al Khalili S, Ndunda N, Petersen E. Cost-effectiveness of IGRA/QFT-Plus for TB screening of migrants in Oman. International Journal of Infectious Diseases. 2020;92:S72-S7.

33. Jo Y, Shrestha S, Gomes I, Marks S, Hill A, Asay G, et al. Model-based cost-effectiveness of state-level latent tuberculosis interventions in California, Florida, New York, and Texas. Clinical Infectious Diseases. 2021;73(9):e3476-e82.

34. Kim H, Hanrahan C, Martinson N, Golub J, Dowdy D. Cost-effectiveness of universal isoniazid preventive therapy among HIV-infected pregnant women in South Africa. The International Journal of Tuberculosis and Lung Disease. 2018;22(12):1435-42.

35. Auguste PE, Mistry H, McCarthy ND, Sutcliffe PA, Clarke AE. Cost-effectiveness of testing for latent tuberculosis infection in people with HIV. AIDS. 2022;36(1):1-9.

36. Fekadu G, Yao J, You JH. Cost effectiveness analysis of single and sequential testing strategies for tuberculosis infection in adults living with HIV in the United States. Scientific Reports. 2022;12(1):1-10.

37. Marx FM, Hauer B, Menzies NA, Haas W, Perumal N. Targeting screening and treatment for latent tuberculosis infection towards asylum seekers from high-incidence countries–a model-based cost-effectiveness analysis. BMC Public Health. 2021;21(1):1-16.

**List of legends**

**Table A.** Search strategy for the primary systematic literature review **Table B**. Search strategy for the secondary systematic literature review (TST and IGRA)

**Table C**. Summary of our results/findings of both reviews

**Table D**. Data extraction results for all the articles found in the primary systematic review

**Table E**. Data extraction results for all the entries from the secondary systematic review

**Table F**. Drummond checklist for studies quality: Cost/Cost-effectiveness analyses for Novel skin tests for diagnosing TBI

**Table G**. Drummond Checklist for studies quality: Cost/Cost-effectiveness analyses for TST or IGRA for diagnosing TBI

**Table H**. Summary of the proportion of articles accomplishing each of the Drummond’s criteria
